# Analysis and identification of coumarins in different parts of *Chimonanthus salicifolius* and biosynthetic pathways prediction

**DOI:** 10.3389/fpls.2025.1656532

**Published:** 2025-10-21

**Authors:** Yu Jiang, Xinhua Ma, Mimi Xu, Tinghui Xie, Yingpeng Tong

**Affiliations:** ^1^ School of Agriculture and Bioengineering, Taizhou Vocational College of Science and Technology, Taizhou, China; ^2^ Zhejiang Hisun Pharmaceutical Co., Ltd., Taizhou, China; ^3^ Institute of Natural Medicine and Health Products, School of Pharmaceutical Sciences, Taizhou University, Taizhou, China

**Keywords:** *Chimonanthus salicifolius*, different parts, UHPLC-ESI-Q-MS/MS, coumarins, biosynthetic pathway

## Abstract

**Introduction:**

*Chimonanthus salicifolius* is a medicine-food homology plant in China with a long application history and various metabolites. However, there is currently a lack of innovative research of analytical approaches on secondary metabolites and integrated study on the chemical compounds of different parts in *C. salicifolius*. It is highly necessary to develop a novel workflow for rapidly screening and identifing metabolites, which will support material basis research for subsequently exploring the applications in fields of food and pharmaceutics of *C. salicifolius*.

**Methods:**

An approach combined identification of in-house library and feature based molecular networking (FBMN) with characteristic fragment ions and neutral losses was employed to analyze the secondary metabolites in the different parts of *C. salicifolius*, with discussion of the diagnostic ion and neutral loss in coumarins and cinnamic acids. And differences in metabolites of different parts of *C. salicifolius* was analyzed by PCA, PLS-DA and volcano plots.

**Results and discussion:**

A total of 200 compounds were identified, of which 69.04% were automatically annotated using self-built R script, effectively accelerating the identification of target compounds, and significantly improving the efficiency of compound structure annotation. The biosynthetic pathway of coumarins was predicted on basis of the identified compounds. Subsequently, a remarkable distinction of metabolites was observed from the shells of seed, leaves and seeds compared to the roots and branches, followed by a relatively minor disparity from branches and roots through PCA analysis and PLS-DA analysis. It was revealed in the heatmap that coumarins, flavonoids, terpenoids, atty acids and cinnnamic aicds were abundant in the leaves of *C. salicifolius*, providing material basis for subsequent pharmacological research.

## 1 Introduction

*Chimonanthus salicifolius* S. Y. (Chinese name “Liu-Ye-La-Mei”) belongs to the genus *Chimonanthus* (Family Calycanthaceae), and is widely distributed in Eastern China, such as Zhejiang province. Its young leaves are the main ingredients of Shiliang Tea, and have been one of traditional national She medicines for hundreds of years. Moreover, *C. salicifolius* receives the title of “the uncrowned king of traditional She nationality medicine” because of their various of definite pharmacological effects (Lv et al., 2020), such as anti-oxidant, anti-bacterial (Wu et al., 2017), anti-inflammatory (Yang et al., 2012), anti-tumor (Liu et al., 2011), anti-diarrheal (Wen et al., 2013), anti-gastrointestinal mucositis (Liu et al., 2013) and so on.

It has been reported in previous literatures that *C. salicifolius* contains rich chemical components, including coumarins (Wang et al., 2016), terpenoids (Zhang, 2013), alkaloids (Ma et al., 2015), flavonoids (Ke, 2010), steroids (Li et al., 2016), quinones (Pan et al., 2012) and others. Coumarins, as the naturally oxygen heterocyclic compounds with multi-bioactivity, are the main active compounds of *C. salicifolius*. There are over 1300 identified natural coumarins from plants and microorganism, which are mainly classified into seven types on basis of their chemical structure, including simple coumarins, bicoumarins, phenylcoumarins, furanocoumarins, pyranocoumarins, isocoumarins and other coumarins (Sun et al., 2020). As far, 28 coumarins isolated from genus *Chimonanthus* not only belong to the first three types of coumarins mentioned above (shown in **Supplementary Figure S1**), but also include coumarin trimers and coumarinolignans, which reveals that its coumarins are rich in variety. And the literature review indicates that natural chimsalicifoliusins is only found in genus *Chimonanthus*. It also illustrates that study of coumarins in *C. salicifolius* displays certain scientific significance.

The main biosynthetic pathways of some simple coumarins have been well characterized (Wang et al., 2025). Coumarins, generally derived from the phenylpropanoid pathway, involve a series of catalytic enzymes, including phenylalanine ammonia lyase, cinnamic 4-hydroxylase, cinnamate 3-hydroxylase, caffeic acid *O*-methyltransferase, and 4’-coumarin-CoA ligase, which induce the production of the key precursor for coumarin formation, cinnamic acid derivatives, such as cinnamic acid, *p*-coumaric acid, *p*-coumaroyl CoA, caffeic acid and ferulic acid (Bourgaud et al., 2006; Li et al., 2024). And cinnamic acid derivatives form simple coumarins under the action of some catalytic enzymes, like feruloyl-CoA 6’-hydroxylase, cinnamate 2’-hydroxylase, coumarin synthase, scopoletin 8-hydroxylase, laying the foundation for the subsequent biosynthesis pathways of specific branches of coumarins (Bai et al., 2024; Wang et al., 2025). For example, umbelliferone is a simple coumarin as well as an important precursor for the formation of complex coumarins, such as pyranocoumarin or furanocoumarin (Del Río et al., 2014). As known to all, chimsalicifoliusins, the specific components of *C. salicifolius*, belong to bicoumarins. However, research on the biosynthetic pathway of such components remains relatively limited, and further exploration is needed.

Ultrahigh performance liquid chromatography-tandem mass spectrometry (UHPLC-MS/MS) is a highly sensitive and rapid tool, which is widely used for structure elucidation of secondary metabolites from plants, fungi and animals, thus it has drawn focus from the food and pharmaceutical science realm. As an ionization MS, electrospray ionization coupled with quadrupole extractive orbitrap MS (ESI-QE-Orbitrap-MS/MS) can provide accurate precursor and fragment ions data (Venugopala et al., 2013). However, this approach generates a large amount of data owing to thousands of compounds in herb, greatly affecting speed and progress of analysis and research. Therefore, advanced compound screening techniques, like the use of characteristic fragment ions (Matey et al., 2023) and neutral losses (Zhou et al., 2024), have emerged as the times require to enhance and accelerate the structural annotation of target compounds from the huge mass spectra database. The untargeted profiling of coumarins is always difficult because of complicated structures and numerous similar metabolites. Thus, there are currently increasing researches on the analysis and identification of coumarins in various plants by LC-MS/MS technology. For example, LC-MS/MS technology were used to purify and isolate sesquiterpene coumarins from *Ferula assa-foetida* (Chayan et al., 2026). And the technology was applied on simultaneous quantitative analysis and pharmacokinetic analysis of furanocoumarins in *Radix Angelicae dahuricae*, which demonstrated the distribution and accumulation of coumarins in roots (Gao et al., 2022; Chen et al., 2015). Simultaneously, furanocoumarins in grapefruit were also annotated with a rapidly profiling strategy integrating LC-MS and mass data filter tool MassQL with molecular networking (Gong et al., 2025). However, there are few studies analyzing chemical constituents, especially coumarins in *C. salicifolius* by LC-MS/MS, even in genus *Chimonanthus* (Zhang et al., 2009; Chen et al., 2017).

The fundamental preliminary step in compound screening just is identifying the characteristic fragment ions and neutral losses of the target compound, which supplies an important reference point for the subsequent structural annotation of the corresponding compounds. Nevertheless, it is still necessary to overcome difficulties in the use of characteristic fragment ions and neutral losses for compound screening. The differences in characteristic fragment ions and neutral losses of the same compound can appear with alteration of experimental instruments and analytical conditions, like the fragment ion at m/z 151 Da, commonly identified as a diagnostic ion for flavonoids in negative mode (Lu et al., 2020), which has not been consistently observed in other experiments. In the meantime, compounds in different types also exhibit the same fragment ions and neutral losses, such as fragment ion at m/z 137 Da, the diagnostic ion for hydroxybenzoic acid (Zhang et al., 2023), catechins (Chen et al., 2023), and quinic acid (Zhang et al., 2023), the simultaneous presence of which may act on significant interference levels in the screened mass spectra data. As a result, it is crucial to systematically analyze characteristic fragment ions and neutral losses related to candidate compound types in various experiments.

Global Natural Product Society (GNPS) method with the web platform (http://gnps.ucsd.edu) has been established since 2014. The mass spectra data can be processed and visualized with construction of a total mass spectra molecular network, in which compounds with same structure units can be clustered into the same sub molecular network to classify compounds with different types (Tong et al., 2021). Therefore, GNPS molecular network has emerged as a convenient and effective approach to greatly simplify the process of data analysis and accelerate analysis speed, that can identify unknown compounds from plant-derived food and medicines effectively and solute problems caused by large amounts of data to some extent.

In the current work, an approach combined identification of characteristic fragment ions and neutral losses with in-house library and GNPS-molecular network was established to applied for annotation of chemical compounds in *C. salicifolius*. Initially, an in-house library was constructed and utilized with GNPS database to screen specific compounds for manual verification. Afterwards, the characteristic fragment ions and neutral losses of related compounds in *C. salicifolius* were identified through the MS2 spectra of identified compounds, which were regarded as the key link of screening process. Then, the selection of various compound types in *C. salicifolius* was determined via the screening process and the compound structures were annotated through visualization mass spectra network, among which several coumarins were get from *C. salicifolius* for the first time. Additionally, the method was also explored the application on biosynthetic pathway prediction of coumarins, as well as annotation analysis of the intermediate compounds generated in the biosynthetic pathway. Subsequently, a comprehensive evaluation of diverse secondary metabolites in different parts of *C. salicifolius* would be achieved by means of the application of Principal Component Analysis (PCA), Partial Least Squares Discriminant Analysis (PLS-DA), and additional analytical methodologies. This study aimed to investigate the effective compounds in different part of *C. salicifolius* through a speedy and effective approach which will support further investigation for plant-derived secondary metabolites analysis.

## 2 Materials and methods

### 2.1 Materials and sample preparation for UHPLC-MS/MS

The leaves, branches, roots, seeds, shells of seed and spray-dried powder of *C salicifolius* were collected from Lishui, Zhejiang Province in China, located at 28°N and 119°E, where the GAP (Good Agricultural Practice) planting bases of *C. salicifolius* were built. The local climate is warm and humid all year round with simultaneous rain and heat. And the soil environment is slightly acidic or neutral fertile soil. The raw materials were dried in a hot air oven at 50 °C. And the dried materials crushed into powder at high speed in a micronizer, which were sieved through a 50-mesh sieve. In addition, Subsequently, 1.0 g dried powders of leaves, branches, roots, seeds, shells of seed and spray-dried powder were precisely weighed and placed in a 50 mL centrifuge tube respectively, mixing with 20 mL of 70% methanol. The mixtures were extracted using ultrasonic assisted extraction method at a constant temperature of 50 °C for a duration of 30 min. Then, the supernatants were taken after separation by centrifugation at 6000 g/min for 10 min and filtered with 0.22 *μ*m microporous filter membranes to get samples for UHPLC-MS/MS analysis. The samples were preserved in a 4 °C refrigerator temporarily for subsequent use.

LC-MS grade chemical reagents, including formic acid, methanol, acetonitrile, were all purchased from Sigma-Aldrich (St. Louis, USA). The ultrapure water was provided from Wahaha Corporation (Hangzhou, China) and formic acid was added to ultrapure water as one of the mobile phases for chromatographic analysis.

### 2.2 UHPLC-Q-Orbitrap-MS/MS analysis methodology

The prepared samples were subjected to analysis by a Thermo UHPLC-MS/MS system composed of an Ultimate R3000 UHPLC (Thermo Fisher Scientific, an ACQUITY UPLC HSS T3 column, 1.8 *μ*m, 2.1×100 mm, Waters Corporation, Milford, CT, USA) with a diode array ultraviolet light detector (UV-DAD) coupled to an Exactive™ MS (Thermo Scientific™, Sunnyvale, CA, USA) in positive and negative ion mode.

The mobile phase system consisted of solvent A (MeCN) and solvent B (0.5% formic acid/water solution, V/V). The oven temperature was maintained at 30 °C with injection volume of 2 *μ*L. And the flow rate was kept at 0.3 mL/min and DAD detection wavelength was set at 236, 254, 300 and 360 nm. The gradient elution condition was as follows: 0-12.5 min, 95-75% B; 12.5–25 min, 75-55% B; 25-37.5 min, 55-50% B; 37.5–50 min, 50-40% B; 50-50.1 min, 40-95% B; 50.1–60 min, 95% B. The heated electrospray ion source (HESI) was used as ion source in MS system, and its heater temperature was 350 °C. The mass and UV spectra were acquired using Xcalibur 4.2 software (Thermo Fisher Scientific, California, USA).

### 2.3 Feature-based molecular networking through GNPS

The MS/MS data were acquired with UHPLC-MS/MS analysis according to the abovementioned conditions. The RAW files of mass spectrometry data were converted to.mzXML format using GNPS_Vendor_Conversion software from GNPS website. The data with.mzXML format were imported to MZmine 2.5.3 and processed for chromatographic feature extraction (Pluskal et al., 2010). The noise levels for MS1 and MS2 in MZmine filter parameters were set at 1106 and 1103, respectively. The MS feature quantification table (.CSV file) with peak areas and MS2 spectral summaries (.MGF file) with representative MS2 spectra were exported from MZmine software (Nothias et al., 2020). Then, the CSV files and MGF files were subjected to GNPS online platform (https://gnps.ucsd.edu/) and analyzed on basis of Feature-based Molecular Networking (FBMN) to complete MolNetEnancer process (Jiang et al., 2022). The preliminary annotation of metabolites in *C. salicifolius* was completed through comparisons of spectra of nodes in FBMN against GNPS public mass spectral library. The matches between node spectra and library spectra were filtered to get a cosine score above 0.6 and at least 5 matched peaks. The FBMN were visualized by Cytoscape 3.9.1 (Shannon et al., 2003).

### 2.4 Construction of an in-house library of metabolites from genus *Chimonanthus* plants

Considering the relatively limited studies on metabolites in *C. salicifolius*, the investigation collected metabolites from genus *Chimonanthus* plants for systematically analysis. For construction of the in-house library to aid dereplication, we gathered previously available literature on phytochemistry of the only six plants of genus *Chimonanthus*, including *C. salicifolius*, *C. zhejiangensis*, *C. praecox*, *C. grammatus*, *C.nitens* and *C. campanulatus*, by searching on the foreign language databases and Chinese databases like Scifinder^®^, Web of Science and CNKI databases. Meanwhile, the reported compounds were summarized and classified according to their structure types. In addition, components with high matching confidence scores (>0.9) in the GNPS library were also included in the in-house library. The chemical structures of compounds in the in-house library were redrawn in chemBioDraw Ultra 14.0 software, and validated through PubChem and Scifinder websites with adding molecular formulas, exact masses and [M+H]^+^ ions. Duplicate compounds were removed from the database. A spreadsheet was created with information regarding detail of coumarin and cinnamic acid compounds presented in **Supplementary Table S1** (**Supplementary Material**).

### 2.5 Identification of chemical compounds from *C. salicifolius*

A self-built R package was developed for automatic screening to achieve rapid identification of metabolites in *C. salicifolius.* The detail code was presented as **Appendix I** in the **Supplementary Material**. The self-built R package was used to record the number of matched MS^2^ ions of each component in the screening process. And the nodes of target compounds with over 60% characteristic ions were further identified through integrating the neutral losses of various compounds.

Moreover, it was necessary to carry out manual verification in combination with FBMN for the automatic identification results due to the confirmation of only substructures in some nodes. The known chemical constituents were dereplicated and new molecules were identified through the performance of molecular network analysis and manual interpretation of MS/MS spectra (Peres et al., 2023). The diagnostic ion fragments were compared with data from literature to identify chemical compounds in *C. salicifolius* tentatively on basis of in-house library. If there were no data of compounds from in-house library matching ion fragment of UHPLC-MS/MS analysis, possible chemical structural units were retrieved from PubChem, Scifinder and GNPS database, which could be assembled with the identified substructures, thereby completing the identification of potential compounds.

### 2.6 Data statistical analysis

The retention time (RT), accurate molecular weight (m/z) and MS/MS fragment ions were collected from the raw data through Xcalibur software (Thermo Fisher Scientific, USA). Then, the collected data were imported into Compound discover 3.2 software (Thermo Fisher Scientific) in RAW format for pre-processing, including denoising, peak alignment, peak recognition, peak feature extraction, overlapping peak analysis, and peak filling, to get the MS feature quantification table (.CSV file). Subsequently, the R packages of ropls was used to analyze the MS feature quantification table for principal component analysis (PCA) and partial least squares discrimination analysis (PLS-DA) (Xu et al., 2024). The MS functions with VIP>1 were considered to be related for group discrimination. The rstatix package was used for one-way analysis of variance (ANOVA) in which MS feature with p < 0.05 and Fold Change (FC) >2 was considered a deferential MS feature (Tian et al., 2024). The combination of PLS-DA and ANOVA analysis was used to screen for MS features with significant differences. In this study, PCA, PLS-DA, and volcano plots were visualized using the R package of ggplot2 and ggprism.

## 3 Results

### 3.1 The base peak chromatograms of *C. salicifolius* samples

**Figure 1:**
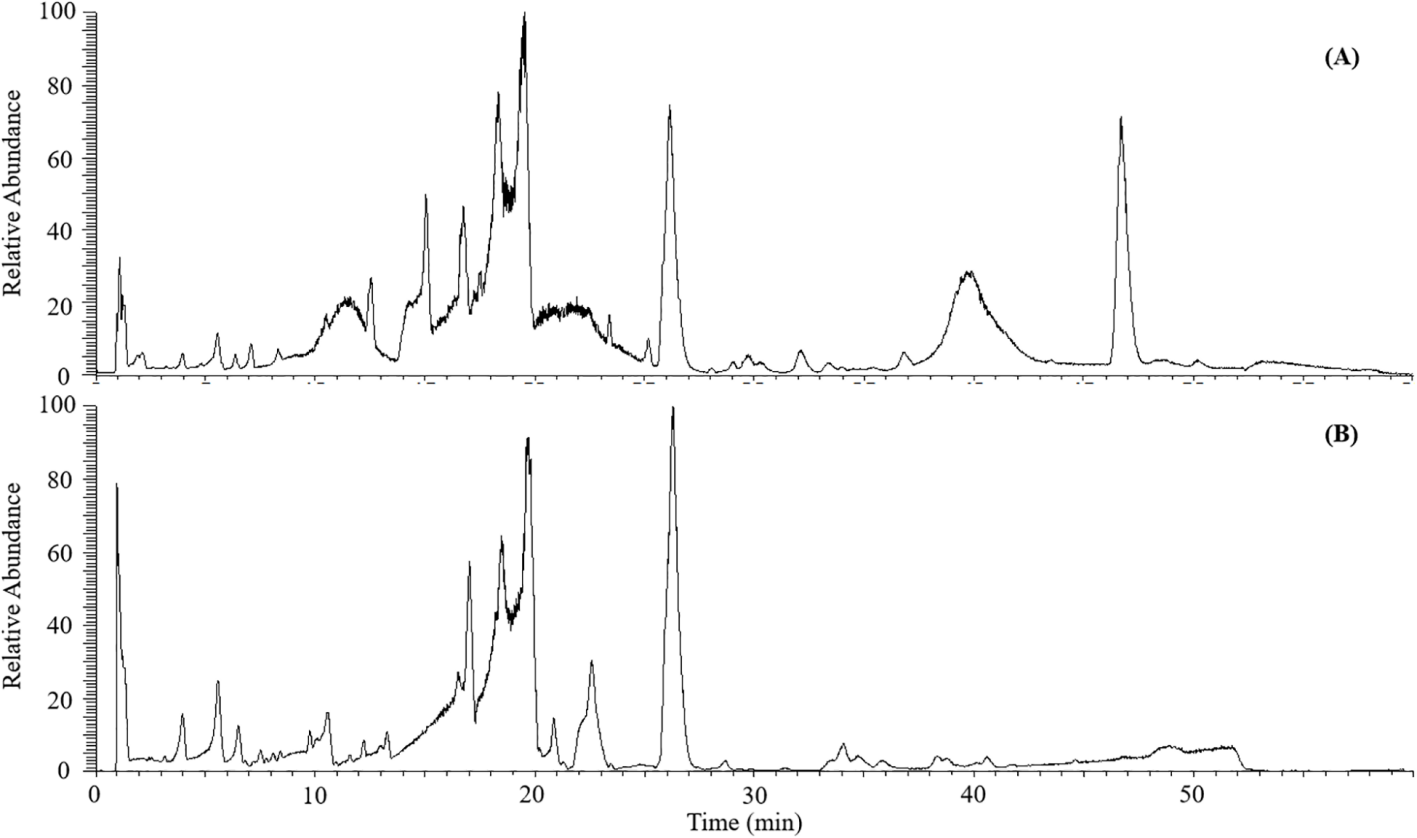
The base peak chromatograms of leaves from *C. salicifolius* in the positive **(A)** and negative **(B)** ionization mode.

The mass spectra of *C salicifolius* samples in negative and positive ionization mode were obtained to better study their potential chemical constituents, taking the leaves from *C salicifolius* as an example, as shown in **Figure 1**. As known, medicinal plants are complex materials composing of thousands of chemical compounds with various structures. Considering this condition, the method with a long elution gradient (60 min) was proposed, which could make certain that all target constituents have a good acquisition. And matrix effect is mitigating through the early elution of matrix compounds along with the decreasing co-elution of target compounds, which ameliorates the identification process (Tahri et al., 2016). It was revealed in **Figure 1** that signal response was more sensitive to majority ingredients in positive ionization mode with comparison of base peak chromatogram in the negative ionization modes. Meanwhile, it could be found that the positive ionization mode was employed to most coumarins analysis based on literature research (Zia-ur-Rehman et al., 2022; Liu et al., 2022). Thus, the subsequently structural annotation study on metabolites in *C salicifolius* on basis of mass spectrometry data acquired in positive ionization mode was conducted in this work.

### 3.2 Description of feature based molecular network

As illustrated in **Supplementary Figure S1**, the feature based molecular network was constructed from the positive ionization mode data encompassed a total of 7653 precursor ions, which distributed among 1002 clusters (nodes ≥ 2) and 1465 nodes. Compounds with identical ion fragments possess similar structures, which can be grouped into same molecular cluster in MN according to the report of literature (Quinn et al., 2016). In the GNPS MN analysis, the chemicals were mainly divided into more than six structure types, including flavonoids, coumarins, cinnamic acids, carboxylic acids, phenols, fatty acyls and so on. Nodes were connected by edges to form clusters in same molecular family, and the nodes at the bottom of MN were not clustered. A total of 92 nodes with deduplication and high similarity scores (>0.9) were matched to corresponding components through comparison with GNPS public mass spectral library.

**Figure 2:**
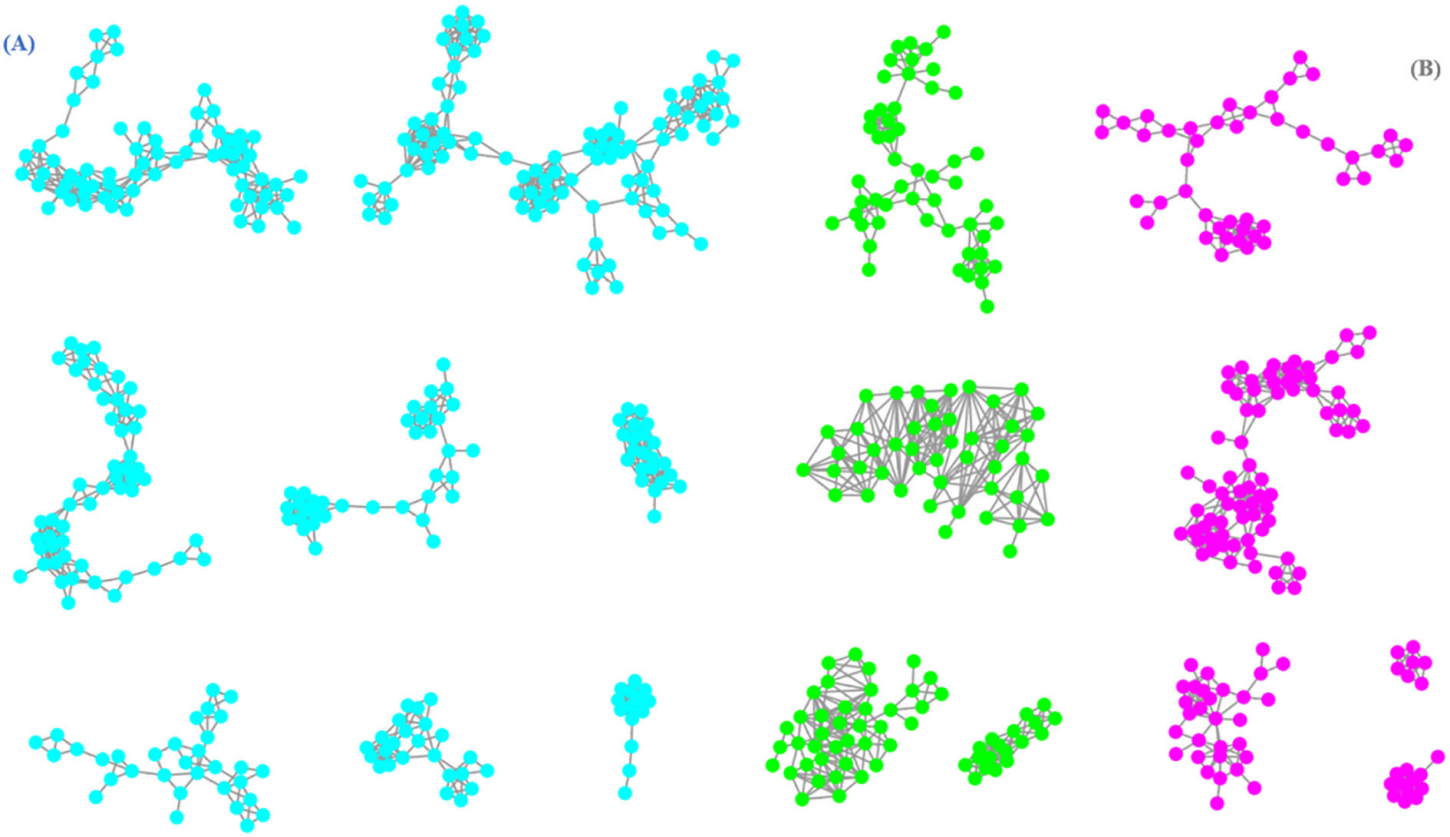
The molecular network of coumarins **(A)** and cinnamic acids **(B)** from *C. salicifolius* (clusters in blue color represented coumarins, clusters in red color represented cinnamic acids, clusters in green color represented co-existence of coumarins and cinnamic acids).

Through FBMN analysis, it was been illustrated in **Figure 2** that there were over eight clusters annotated for coumarins (clusters in blue color) and over five clusters cinnamic acids (clusters in red color) which are closely related to biosynthetic pathway of coumarins from total molecular network for subsequent analysis. Meanwhile, it was found that there were four molecular clusters in green color were simultaneously connected with coumarin and cinnamic acid components, which was probably resulted from their structural similarity and similar mass spectrometry data.

### 3.3 Construction and analysis of in-house library for compounds from genus *Chimonanthus*

197 non-repeat natural compounds were collected by retrieval and collation of relevant literature and reported original from genus *Chimonanthus* previously, of which 29 coumarins and 12 cinnamic acids were comprised in the in-house library. Additionally, 17 extra coumarins and 9 extra cinnamic acids were matched and harvested through data analysis from GNPS public mass spectral library, in which unique coumarins and cinnamic acids were merged to supplement information for current in-house library. Thus, the in-house library consisted of 33 coumarins and 16 cinnamic acids due to the presence of partial coumarins and cinnamic acids in both literature retrieval and GNPS library simultaneously. In accordance with the annotation results at each level of ClassFire, compounds with same structural type may be distributed in one or more structural types. At superclass level, coumarins from genus *Chimonanthus* mainly distributed in Phenylpropanoids and polyketides as well as Lignans, neolignans and related compounds. At class level, these coumarins distributed in Coumains and derivatives as well as Coumarinolignans. At subclass, these coumarins mainly distributed in Hydroxycoumarins and Coumarin glycosides. Although an in-house library has been constructed in this paper, the data information on coumarins from *C. salicifolius* was still insufficient in the database.

### 3.4 Fragmentation patterns of the main types of compounds in *C. salicifolius*

#### 3.4.1 Manual confirmation of compounds filtered by in-house library

A total of 115 nodes were filtered out in FBMN through matching the precursor ions and [M+H]^+^ ions of compounds in the in-house library. These compounds covered various types of chemical structures, including flavonoids, coumarins, cinnamic acids, benzoic acids, benzene derivatives, alkaloids, fatty acids and others. There were no terpenoids and steroids found during this process, possibly owing to their low abundance in *C salicifolius*.

However, 28 out of 115 nodes were matched two or more compounds from the in-house library, which can be resulted in the presence of structural isomers from *C salicifolius*, such as flavonoids with different glycosides (luteoloside and quercitrin), isomers with different chemical categories (arteminorin A and 3,3’-biisofraxidin). As a result, it was necessary to manually confirm the chemical structures of matching nodes through comparing the MS2 spectra from reference or Massbank. During the manual annotation process, it was found that 5 out of 28 nodes were coumarins, whose structures were determined by comparing and matching with in-house library and Pubchem. It could be concluded that the matching result of only 2 nodes was not entirely correct, indicating that construction of in-house library is a quite efficient approach for annotating chemical structures of plant metabolites.

#### 3.4.2 Diagnostic ions and neutral losses of coumarins

**Figure 3:**
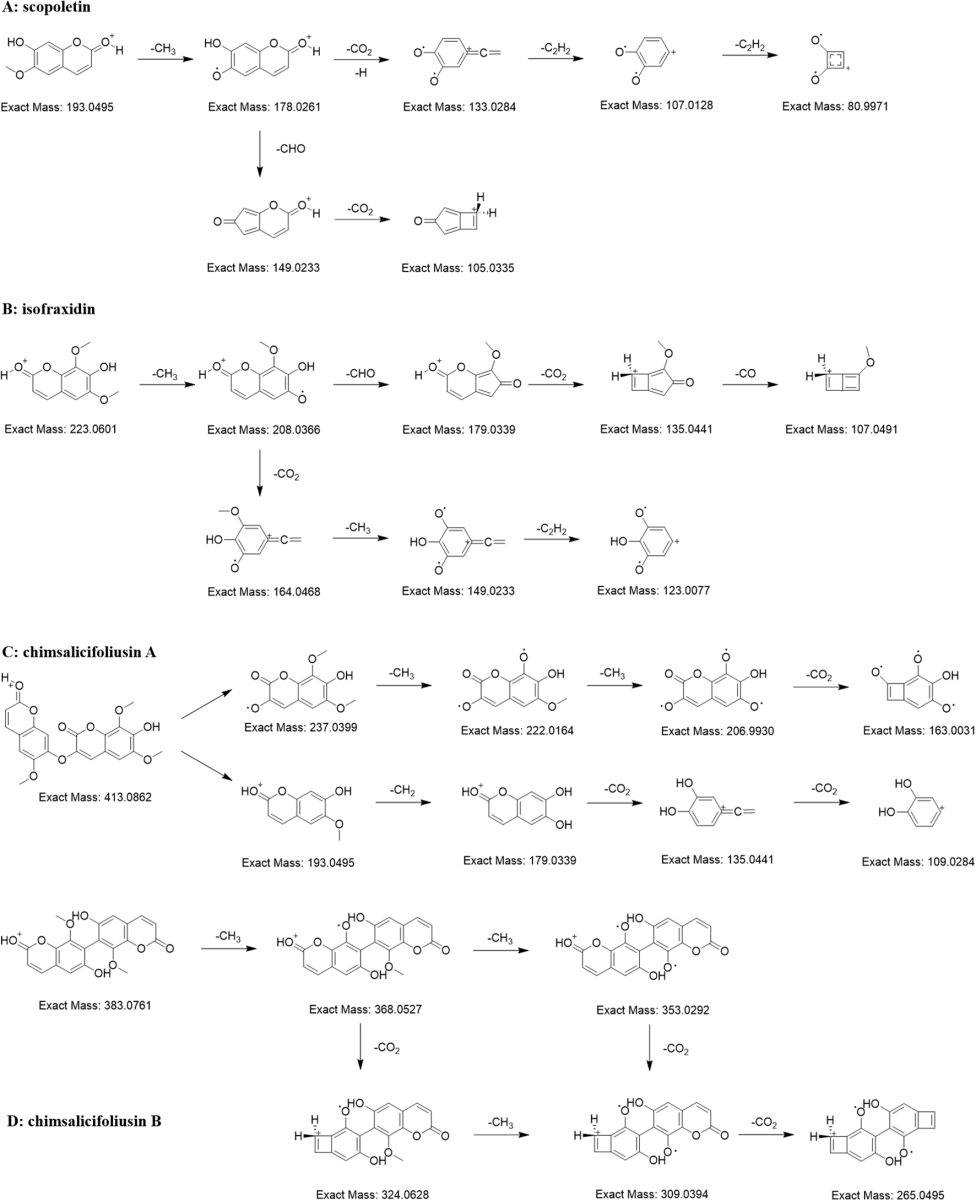
Proposed MS fragmentation pathway for the [M+H]^+^ ion of coumarins [scopoletin **(A)**, isofraxidin **(B)**, chimsalicifoliusin A **(C)** and chimsalicifoliusin B **(D)**].

Coumarins, compounds with basic skeleton of *α*-benzopyranone nucleus, are one of the largest groups of chemical substances from *C. salicifolius*. Through matching with compounds from in-house library in conjunction with manual confirmation, a total of 38 coumarins were annotated in the FBMN of *C salicifolius*. The precursor ion of 34558 was at m/z 193.05 Da and their abundant ions included m/z 178.03 Da ([M+H-CH_3_]^+^), m/z 149.02 Da ([M+H-CH_3_-CHO]^+^), m/z 105.03 Da ([M+H-CH_3_-CHO-CO_2_]^+^), m/z 133.03 Da ([M+H-CH_3_-CO_2_]^+^), m/z 107.01 Da ([M+H-CH_3_-CO_2_-C_2_H_2_]^+^), and m/z 81.00 Da ([M+H-CH_3_-CO_2_-2C_2_H_2_]^+^) (**Figure 3A**), which were consistent with scopoletin (Marzouk et al., 2023). The precursor ion of 34567 was at m/z 223.06 Da and their abundant ions included m/z 208.04 Da ([M+H-CH_3_]^+^), m/z 179.03 Da ([M+H-CH_3_-CHO]^+^), m/z 135.04 Da ([M+H-CH_3_-CHO-CO_2_]^+^), m/z 107.05 Da ([M+H-CH_3_-CHO-CO_2_-CO]^+^), m/z 164.05 Da ([M+H-CH_3_-CO_2_]^+^), m/z 149.02 Da ([M+H-2CH_3_-CO_2_]^+^) and m/z 123.01 Da ([M+H-2CH_3_-CO_2_-C_2_H_2_]^+^) (**Figure 3B**), which were consistent with isofraxidin (Li et al., 2020). It could be concluded that the characteristic ions of coumarins are the neutral losses of CH_3_, CHO, C_2_H_2_, CO and CO_2_ (Concannon et al., 2000).

The precursor ions of nodes 4248 and 4036 were m/z 413.09 Da and m/z 383.08 Da, respectively. They both showed apparent neutral losses of CH_3_ and CO_2_, evidencing that they were potential coumarin dimers. However, there were obvious differences in the MS2 spectra of the two nodes. The abundant ions of 4248 were the ions formed by the continuous neutral losses of CH_3_, CH_2_ and CO_2_ (**Figure 3C**). In the meantime, the abundant ions of 4036 were similar with single coumarin (**Figure 3D**). As is known to all, the difficulty of bond cleavage varies among different chemical bonds, such as the greater difficulty of C-C bond cleavage compared to C-O bond cleavage. Therefore, it could be deduced that 4036 was a coumarin dimer connected by C-C bonds, namely chimsalicifoliusin B, while 4248 was a coumarin dimer connected by C-O bonds, namely chimsalicifoliusin A.

It could be shown in the fragmentation pathways of coumarins that characteristic ions of coumarins were the cleavage ions formed by the loss of neutral molecules, such as CH_3_, CO, CO_2_, from precursor ions. Nevertheless, there were numerous inconsistent fragment ions on account of the different orders of neutral losses, which resulted in the challenging issue of screening coumarins on basis of a small amount of characteristic ions. As illustrated from literature, the distribution of neutral losses of CH_3_, CO and CO_2_ in coumarins overlapped massively with flavonoids and cinnamic acids, which resulted that it was impossible to screen coumarins directly by neutral losses. Thus, the approach to screen potential coumarins could only be achieved through simultaneous analysis of combining precursor ions with neutral losses.

#### 3.4.3 Diagnostic ions and neutral losses of cinnamic acids

**Figure 4:**
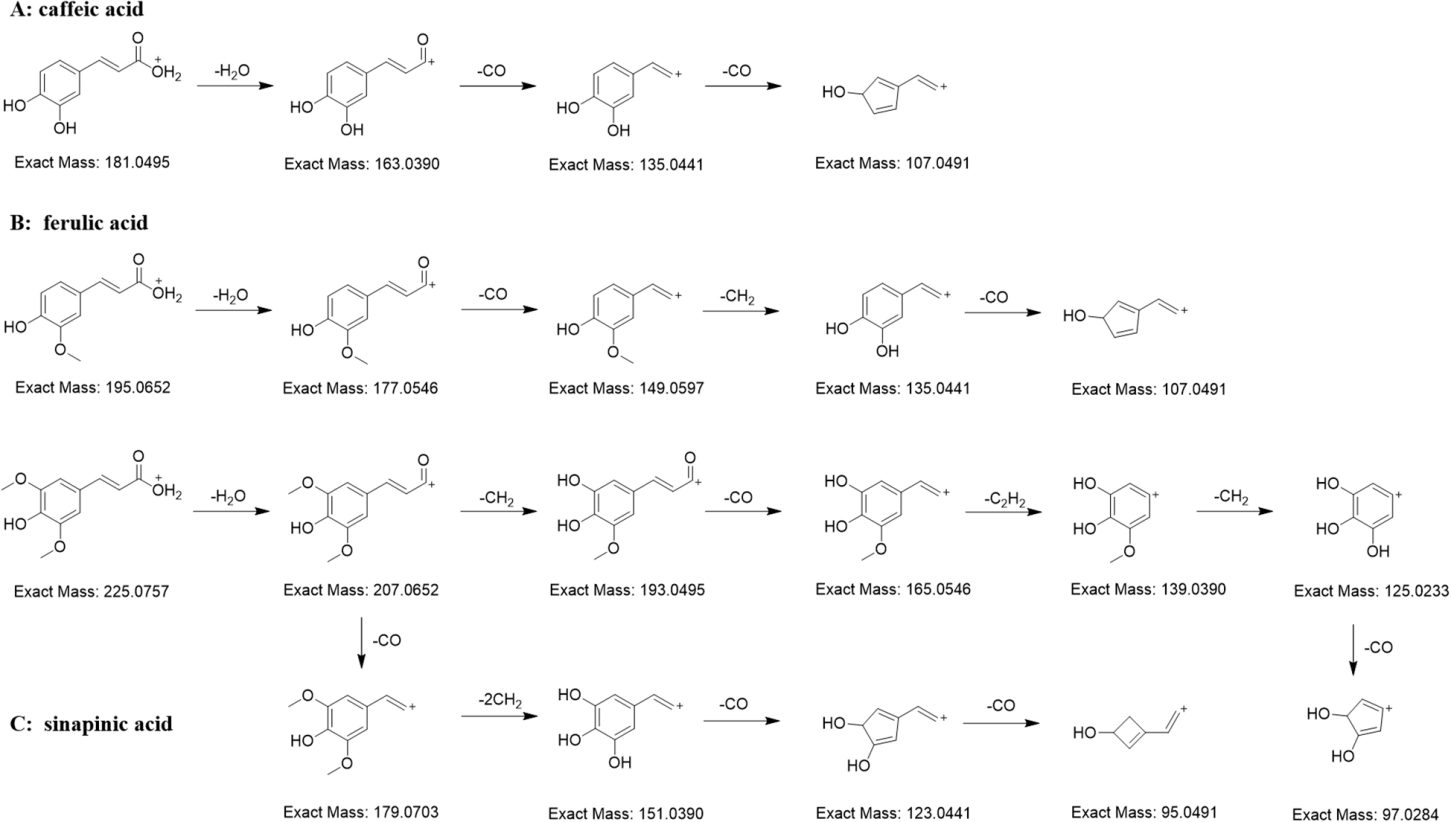
Proposed MS fragmentation pathway for the [M+H]^+^ ion of cinnamic acids (caffeic acid **(A)**, ferulic acid **(B)**, and sinapinic acid **(C)**).

There are many organic acids in *C. salicifolius*, one of which are cinnamic acids, the important components in the biosynthetic pathway of coumarins. A total of 19 cinnamic acids were annotated in the FBMN on basis of matching results by combining in-house library with manual verification. The structural units of cinnamic acids identified from *C. salicifolius* contained caffeic acid (12800), ferulic acid (19207), and sinapinic acid (62459). It could be observed that the ions with relatively high intensities mainly were the ions of [cinnamic acid+H-H_2_O]^+^ and [cinnamic acid+H-CO]^+^, and the intensities of [cinnamic acid+H-H_2_O]^+^ ions was commonly higher than that of [cinnamic acid+H-CO]^+^ ions (**Figure 4**). The cinnamic acid compounds with methoxy groups, ferulic acid and sinapinic acid, were subjected to loss of 15 Da because of the presence of OCH_3_ substituents.

### 3.5 Annotation results of compounds from *C. salicifolius*

A workflow for screening coumarins and organic acids was developed based on the analysis of the structural characteristics of the primary compounds in *C. salicifolius*. The initial screening of target compounds was performed with their characteristic ions and the further analysis was conducted for compounds exhibiting higher likelihoods (score > 0.6). Subsequently, the disparity between the [M+H]^+^ ions of annotated structural unit and its precursor ions was examine, based on which the corresponding structural units were derived. Meanwhile, there might be multiple potential compounds corresponding to a single node, requiring additional manual verification. The compounds that could not be automatically verified were assessed with chemical formula determining through the difference examination between the [M+H]^+^ ions of annotated structural units and their precursor ions. And the appropriate structural units were selected from PubChem database on basis of the calculated chemical formula, with the priority given to compounds with structural similarity to those in the in-house library.

In addition, to identify coumarins, potential nodes related to predicted coumarins were selected on basis of [M+H]^+^ ions via comparing MS1 and MS2 datasets (<10ppm), with the subsequent screening of analyzing neutral losses of CO and CH_3_. Afterwards, the further identification process followed the same protocol as organic acids. The filtering and annotation results are shown in **Supplementary Table S2**.

A total of 38 potential coumarins nodes, were identified in this study through the above-mentioned screening methodology. There was a significant overlap among the screening target compounds based on intersection analysis, such as 15 intersecting nodes between cinnamic acids and quinic acids, with quinic acid coexisting with cinnamic acid structural units. The automatically screening results were annotated onto nodes in FBMN, that could cluster compounds of same types together and observe intersections among numerous chemical structure types, thereupon then giving a benefit to verifying and identifying their structure. As a result, the structure identification of 136 nodes within FBMN was conducted utilizing automatic screening and identification using R language code, dominantly accelerating the compound identification process. Furthermore, the amount of identified compounds were 29 and 32, respectively, through matching with the in-house library and PubChem.

### 3.6 Biosynthetic pathway of coumarins in *C. salicifolius*

There are few reports on the biosynthetic pathway of coumarins in *C. salicifolius.* The phenylpropanoid pathway is one of the most highly recognized biosynthetic pathway of biosynthetic pathway of secondary metabolites in plants, which surely applies to the biosynthesis of coumarin (Wang et al., 2025). Glucose is the primary source for forming phenylalanine and tyrosine through shikimate pathway, which belong to phenylalanines and used as the initial substrate of phenylpropanoid pathway. Phenylalanine ammonia-lyase (PAL) and tyrosine amino lysae (TAL) are catalytic enzymes that convert phenylalanine into trans-cinnamate through non-oxidative deamination reactions, thereby directing the carbon flow of the shikimate pathway to various branches of general phenylpropanoid metabolism (Thomas, 2010). Cinnamic acid and p-coumaric acid just get converted on the action of PAL and TAL, respectively (Sharma et al., 2019; Liu et al., 2014). Simultaneously, cinnamic acid can convert into *p*-coumaric acid by the action of cinnamate 4-hydroxylase (C4H). Both of them are the precursors of downstream metabolic pathway, forming a complex multi branched phenylpropanoid biosynthetic pathway, in which the coumarin biosynthetic pathway is an important branch (Dong and Lin, 2021).

On one hand, caffeic acid and ferulic acid are sequentially formed through the action of coumarate 3-hydroxylase (C3H) and *O*-methyltransferase (OMT) by ortho-hydroxylation and methylation of cinnamates. Methylation reactions are usually catalyzed by O-methyltransferases, which can be divided into caffeoyl coenzyme O-methyltransferase (CCoAOMT) and caffeic acid O-methyltransferase (COMT). The former OMT mainly participates in the methylation of pyrocatechols containing meta hydroxyl groups, while the latter participates in the methylation of pyrocatechols containing para hydroxyl groups (Lam et al., 2007). Considering that 6,7-dimethoxycoumarin was also identified in *C. salicifolius*, it was speculated that more than one OMT were involved in the methylation reaction of coumarin. On the other hand, 2,4-dihydroxy-cinnamic acid is also formed from *p*-coumaric acid on the action of coumarate/cinnamate/ferulate 2-hydroxylase. The aforementioned cinnamic acids are all important metabolites related to the phenylpropanoid pathway, in which *p*-coumaric acid, caffeic acid and ferulic acid were mentioned in the compound annotation of *C. salicifolius*.

3-Coumarate CoA ligase (3-CL) and 4-Coumarate CoA ligase (4-CL) can channel the aromatic CoA-esters to different biosynthetic pathways, which might be the most important branching point in the central phenylpropanoid biosynthesis of plants (Thomas, 2010). Ferulic acid gets converted to feruloyl-CoA catalyzed by 3-CL, subsequently forming scopoletin on the action of feruloyl-CoA 6’-hydroxylase (F6’H), cinnamate 2’-hydroxylase (C2’H). Meanwhile, the action of intramolecular lactonization results in the formation of umbelliferone, which induce the formation of esculetin on the action of esculetin synthase. Umbelliferone has been confirmed to be an important precursor in the biosynthetic pathways of pyranocoumarin and furanocoumarin, which can also be speculated to be the precursor to other complex coumarins, including bicoumarins, coumarinolignans and tricoumarins.

**Figure 5:**
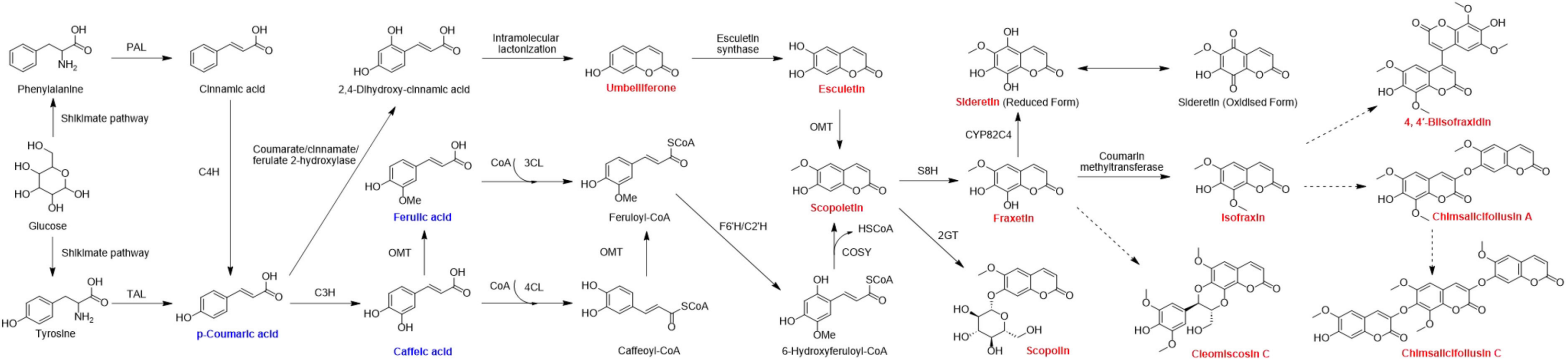
Biosynthetic pathway of coumarins in *C. salicifolius.* PAL, phenylalanine ammonia lyase; TAL, tyrosine ammonia lyase; C3H, cinnamate 3-hydroxylase; C4H, cinnamate 4-hydroxylase; OMT, O-methyltransferase; 2GT, 2-coumarate O-*β*-glucosyltransferse; 3CL, 3-coumarate CoA ligase; 4CL, 4-coumarate CoA ligase; F6’H, feruloyl-CoA 6’-hydroxylase; C2’H, cinnamate 2’-hydroxylase; COSY, coumarin synthase; S8H, scopoletin 8-hydroxylase. The coumarins highlighted in red are the coumarins of *C. salicifolius* that have been analyzed in the manuscript. The cinnamic acids highlighted in yellow are the cinnamic acids of *C. salicifolius* that have been analyzed in the manuscript. Black arrows indicate single direct reaction, while dashed black arrows represent unauthenticated steps.

Subsequently, coumarins are successively formed on the action of different enzymes, including scopolin, fraxetin, isofraxin, as shown in **Figure 5**, all of which were also identified through approach developed in our team. Scopolin and fraxetin are formed catalyzed through 2-coumarate *O*-*β*-glucosyltransferse (2GT) and scopoletin 8-hydroxylase (S8H), respectively. And isofraxin is formed on the action of coumarin methyltransferase. These are known to be involved in simple coumarins skleton formation, whereas the biosynthetic mechanism of complex coumarins, like bicoumarins, coumarinolignans and tricoumarins, remained to be elucidated, which will also be a future research direction.

### 3.7 Differences in metabolites of different parts of *C. salicifolius*

**Figure 6:**
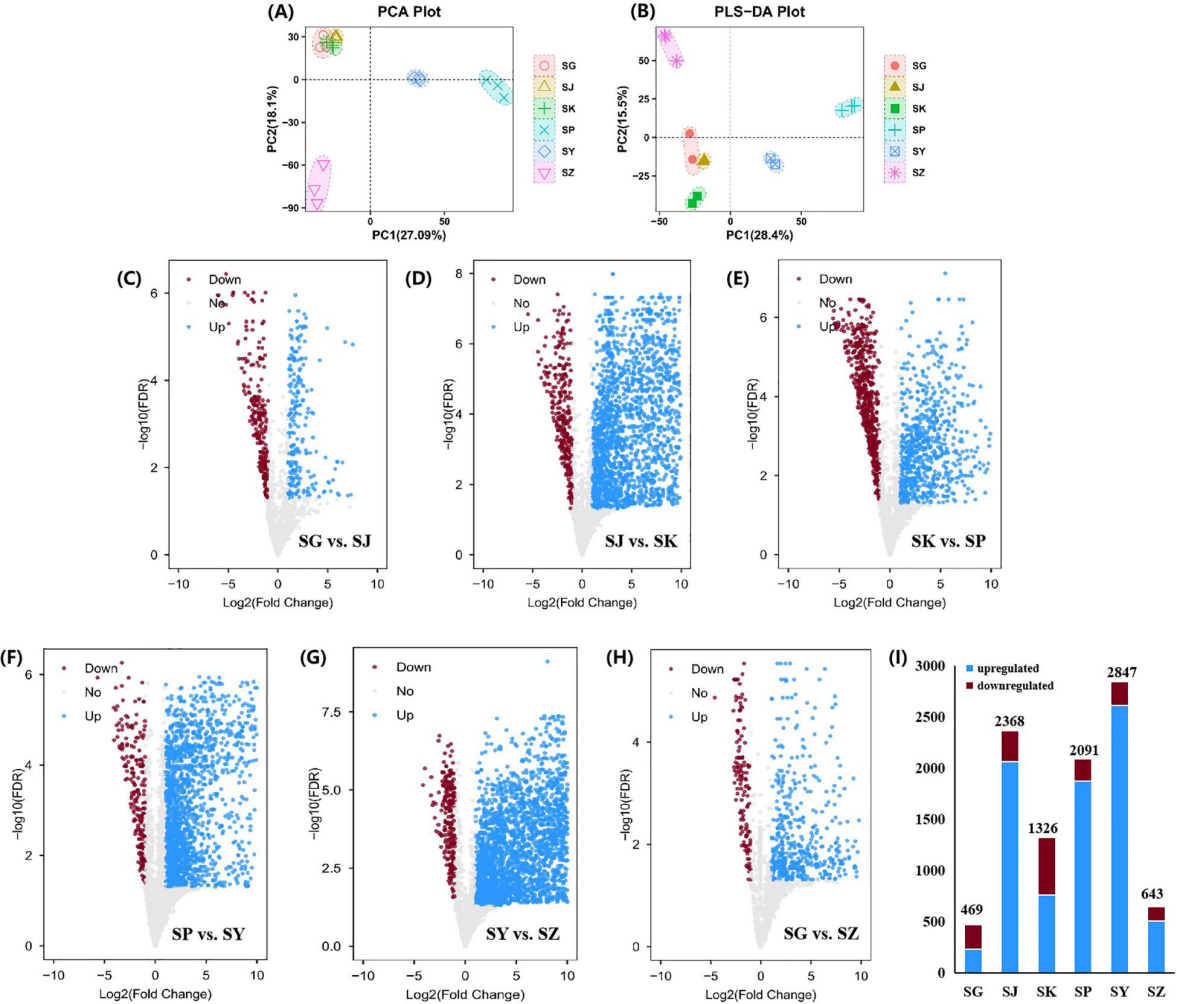
Screening the metabolites with significant difference. **(A)** PCA score plot for *C. salicifolius* samples of different parts. **(B)** PLS-DA score plot for *C. salicifolius* samples of different parts. **(C-G)** Volcano plot of metabolites in samples collected in SG vs. SJ **(C)**, SJ vs. SK **(D)**, SKvs. SP **(E)**, SP vs. SY **(F)**, SY vs. SZ **(G)**, SG vs SZ **(H)**. **(I)** The changes of upregulated metabolites and downregulated metabolites in samples of different parts. (SY:leaves, SJ:branches, SG:roots, SZ:seeds, SK:shells of seed, SP:spray-dried powder).

It was illustrated from the PCA results that there was remarkable separation between samples from different parts of *C. salicifolius* (**Figure 6A**), including leaves, branches, roots, seeds, shells of seed and spray-dried powder, indicating potential variations in their metabolic profiles. Specifically, samples of leaves, seeds and spray-dried powder of *C. salicifolius* exhibited significant intra-group differences, whereas the intra-group variability among branches, roots and shells of seed *C. salicifolius* was relatively minimal. This demonstrated that significant influence was acted on the secondary metabolites of different parts in *C. salicifolius*, thereby introducing heterogeneity within the samples. VIP > 1 was used as a criterion, and a total of 3590 markedly different metabolites was identified through PLS-DA analysis (**Figure 6B**).

Using a fold change (FC) threshold of ≥ 2 and a significance level of P < 0.05 as criteria, analysis with volcano plot was conducted to identify differential metabolites between samples from different parts of *C. salicifolius* (**Figures 6C–H**). The analysis suggested that there were 469 differential metabolites in the roots of *C. salicifolius*, compared with the samples of branches, with 230 metabolites upregulated and 239 downregulated. In the same way, 2368 differential metabolites were observed when branches samples were compared with shells of seed of *C. salicifolius*, with 2060 upregulated and 308 downregulated. Relative to spray-dried powder sample, the shells of seed of *C. salicifolius* exhibited 1326 differential metabolites with 757 being upregulated and 569 being downregulated. There were 2091 differential metabolites in the spray-dried powder sample with comparison of leaves sample, with 1872 being upregulated and 219 being downregulated. And it was observed that there were 2847 differential metabolites in the leaves of *C. salicifolius*, compared with seeds, with 2611 upregulated and 236 downregulated. Additionally, 643 differential metabolites were observed when the roots of *C. salicifolius* were compared with seeds samples, with 507 upregulated and 136 downregulated (**Figure 6I**). The quantity of differential metabolites revealed a significantly higher number of differential metabolites between the samples of branches and shells of seed, leaves and seeds compared to the roots and branches. This suggested that there were significant differences in the metabolic profiles of different parts of *C. salicifolius*.

**Figure 7:**
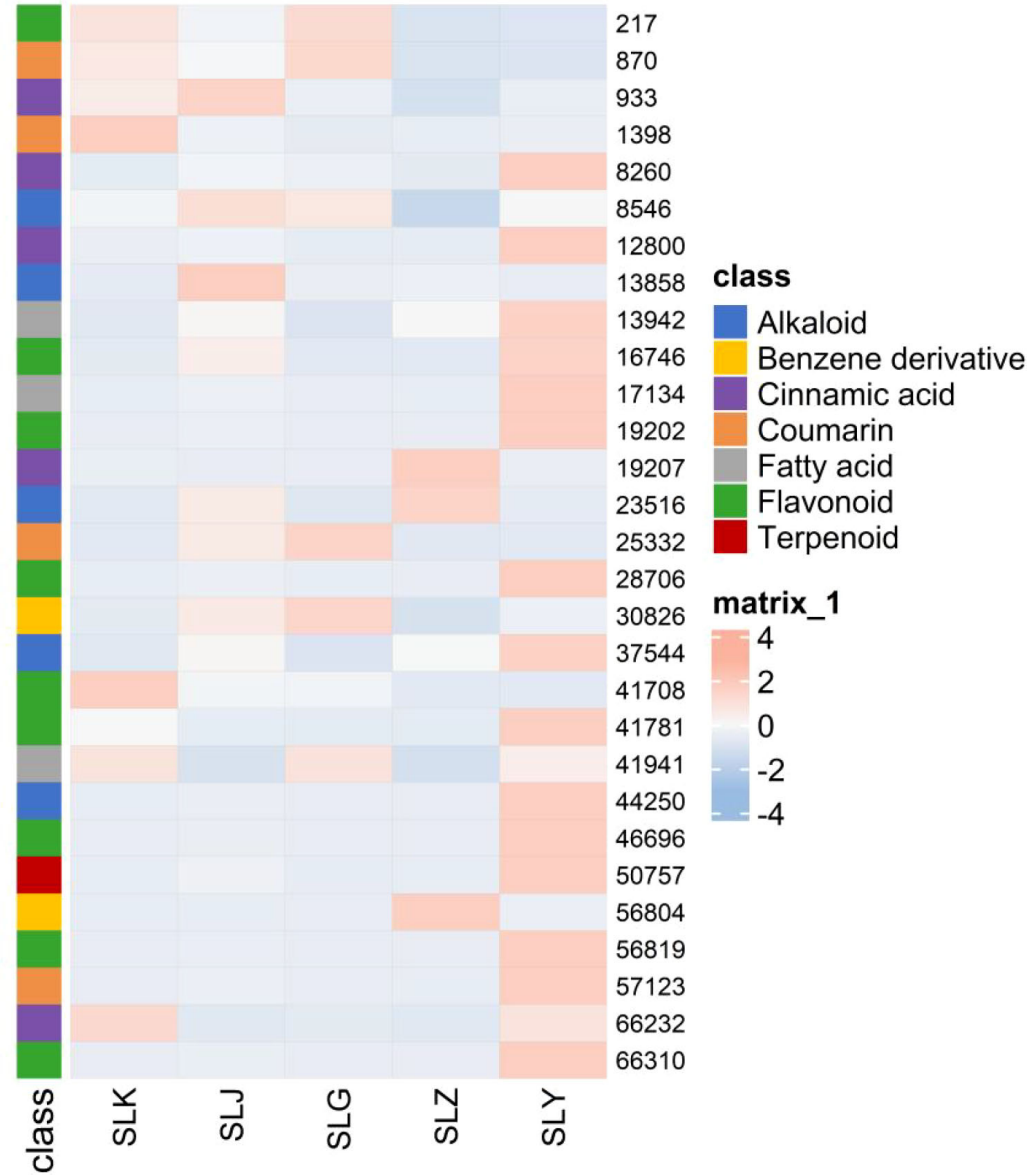
Heatmap of identified metabolites with significant difference.

A total of 1786 differential metabolites were harvested through the cross reference of the results from the volcano plot analysis (P < 0.05) with the PLS-DA analysis (VIP > 1). And 51 metabolites were identified with the follow-up annotation studies of compound structures, including 9 cinnamic acids, 19 flavonoids, 5 coumarins, 4 fatty acids, 9 alkaloids, 3 Benzene derivatives and 2 terpenoids. Significantly, some differential nodes represented different adduct ions of the same compound. For these nodes, the clustered heat map was generated with the retaining of a single adduct ion, to illustrate the differential metabolites in *C. salicifolius* samples of different parts (**Figure 7**). It was revealed in the heatmap that flavonoids, terpenoids, fatty acids and cinnamic acids were most abundant in the leaves of *C. salicifolius*, whereas alkaloids were most prevalent in the branches. The levels of coumarins were found to be higher in shells of seed, roots and leaves. And the levels of benzene derivatives were highest in roots and seeds. These findings provided significant insights into the variations in metabolite content across different parts.

## 4 Discussion

### 4.1 Screening and identification of compounds in *C. salicifolius*

Bioactive secondary metabolites are widely distributed in various medicinal plants, including coumarins, cinnamic acid, flavonoids, phenols, etc (Sharma et al., 2019). Due to the abundant secondary metabolites in medicinal plants, a large amount of data will be generated with analysis by LC-MS/MS, thereby developing new technologies with rapid processing of data (Tong et al., 2021). In this study, an in-house library of identified plant compounds was established and compared with a public mass spectrometry database for the rapid identification of specific chemical components in *C. salicifolius*. Simultaneously utilizing existing literature to identify characteristic fragment ions and neutral losses, and combining them with mass spectrometry molecular network methods, effectively promotes the screening and structural annotation of secondary metabolites in *C. salicifolius*. In this study, these methods, utilizing R language code, accelerate the identification of target compounds, reduce interference from non target compounds, and significantly improve the efficiency of compound structure annotation. During the manual verification process, it was found that there were still inaccuracies in the identification results, which might because of insufficient automatic screening parameters for coumarins.

Coumarin is the main chemical component of *C. salicifolius* with rich biological activities (Wang et al., 2016). Based on literature analysis, it is impossible to only screen coumarin based on a few characteristic ions owing to the different order of neutral loss making many inconsistent fragment ions. Meanwhile, it is also challenging to directly screen coumarins through neutral losses, which results from the significant repetition of neutral loss distribution, such as CH_3_, CO, and CO_2_, in flavonoids, cinnamic acids as well as coumarins (Tong et al., 2025). There were different types of coumarins annotated using the abovementioned analysis method in this study, and the fragmentation pathways of different coumarins, such as simple coumarins and bicoumarins, were explored, from which characteristic ions of coumarins and neutral losses could be observed, contributing to screening and identification of potential coumarins with various types. For example, chimsalicifoliusin A and chimsalicifoliusin B both belong to coumarin dimer with similar structures, being generally difficult to distinguish. However, chimsalicifoliusin A is a coumarin dimer connected through a C-C bond, while chimsalicifoliusin B is a coumarin dimer connected through a C-O bond. The C-O bond is more prone to breakage than the C-C bond, making for exhibition of certain differences in the neutral loss and characteristic ions, which resulted in effectively identification of chimsalicifoliusin A and chimsalicifoliusin B through the analysis approach in this study. However, it was still challenging to screen and identify coumarins on basis of a small number of characteristic ions owing to numerous inconsistent fragment ions from the different order of neutral loss.

### 4.2 Biosynthesis of coumarins in *C. salicifolius*

Several genes, including PAL, TAL, OMT, 3CL, 4CL, C3H, C4H, C2’H, F6’H, 2GT, COSY and S8H, are involved in the biosynthesis of coumarins in *C. salicifolius* for action of methylation, acylation, oxidation, reduction and glycosylation. These genes not only exhibit tissue-specific expression pattern, but also almost act on the general phenylpropane pathway. The regulation of these genes in the synthesis pathways of other phenylpropanoids, such as flavonoids and lignans, in comparison of coumarins, has been more extensively studied, which can provide valuable clues for studying transcriptional regulation of coumarin biosynthetic pathways resulting from that these pathways possess the same phenylpropane metabolic network as biosynthesis of coumarins (Thomas, 2010). For example, CCoAOMT, one of OMT, is the main factor involved in lignin biosynthesis and also plays a role in the biosynthetic pathway of some soluble phenylpropane (Do et al., 2007). The gene expression level of CCoAOMT was significantly increased when subjected UV and low temperature stress, which promoted the accumulation of methylated coumarins in *C. salicifolius* and contributed to formation of generate 6,7,8-trimethoxycoumarin and 5.6,7-trimethoxycoumarin of *C. salicifolius* (Cheng, 2023). F6’H achieved crucial neighboring oxygenation steps to produce of scopoletin through catalyzing the formation 6’-*O*-hydroxylated trans isomer from feruloyl CoA (Kai et al., 2008). The the most prominent soluble coumarins, scopolin, was formed by the modificaion from coumarin aglycones through a set of UDP-glucose-dependent glucosyltransferases. It was found that the involved cinnamic acids, including *p*-coumaric acid, ferulic acid and caffeic acid, have been annotated, indicating that coumarin biosynthesis is closely related to cinnamic acids through the exploration of biosynthetic pathway of coumarins in *C. salicifolius*. There are some reports on the biosynthetic enzymes and pathways of furanocoumarins and pyranocoumarins (Goldenberg et al., 2025), but there is still a lack of reports on the biosynthetic pathway of bicoumarin, the characteristic compound in *C. salicifolius*. Umbelliferone is the entry point for the biosynthesis of furanocoumarin, catalyzed by multiple isoprene transferase (PT) and cytochrome P450 family members, which can serve as a reference for predicting the biosynthetic pathway of bicoumarin in the follow-up research.

### 4.3 Effect of different parts on metabolites from *C. salicifolius*

The biosynthesis, accumulation, and metabolism of secondary metabolites in plants are affected by the regulation of various non biological and biological factors, which induce the significant differences in the types and quantities of metabolites in the different parts of same plant (Sehaki et al., 2023). And the relative metabolites levels of different parts in plants, including bark, branches, leaves, flowers, and fruits, represent the characteristics and distribution of overall nutrition, that are beneficial to explore plant parts with targeted bioactive metabolites as medical resources (Miao et al., 2025). In this study, a total of 1786 differential metabolites were collected using PLS-DA analysis and volcano plot analysis, indicating that there were significant differences in secondary metabolites in different parts of *C. salicifolius* (leaves, branches, roots, seeds, seed shells and spray dried powder), especially coumarins, terpenoids, cinnamic acid, flavonoids and other components. It was shown in the heatmap that the levels of flavonoids, terpenoids, and cinnamic acids were highest in the leaves, whereas coumarins were abundant in shells of seed, roots and leaves, which might be related to the different endophytic flora and environmental conditions in different parts, which can regulate biosynthetic pathways to induce differences in metabolite accumulation (Qu et al., 2022).

## 5 Conclusion

This study comprehensively investigated the secondary metabolites in different parts of *C. salicifolius* through mass spectrometry data analysis and metabolomics. A total of 38 coumarins and 19 cinnamic acids were identified and the biosynthetic pathway of coumarins were predicted based on analysis results of metabolites. Multivariate statistical analysis revealed that there were significant differences in secondary metabolites from different parts of *C. salicifolius.* The levels of bioactive compounds, cinnamic acids, coumarins and flavonoids were found to be highest in leaves, which indicated that leaves might be the most pharmacologically active part of *C. salicifolius*, providing material basis for subsequent researches.

## Data Availability

The original contributions presented in the study are included in the article/**Supplementary Material**. Further inquiries can be directed to the corresponding author.
