# Supplementary material for: Analysis and identification of coumarins in different parts of Chimonanthus salicifolius and biosynthetic pathways prediction
Source: Front Plant Sci. 2025 Oct 21;16:1656532. doi: 10.3389/fpls.2025.1656532 (PMC12583175; doi:10.3389/fpls.2025.1656532)
Supplement: Supplementary file 1 [file DataSheet1.docx]

**Supplementary Material**

Yu Jiang^1^, Xinhua Ma^2^, Mimi Xu^2^, Tinghui Xie^1^, Yingpeng Tong^3*^

1 School of Agriculture and Bioengineering, Taizhou Vocational College of Science & Technology, Taizhou, China

2 Zhejiang Hisun Pharmaceutical Co., Ltd., Taizhou, China

3 Institute of Natural Medicine and Health Products, School of Pharmaceutical Sciences, Taizhou University, Taizhou, China

*Correspondence: Yingpeng Tong, fish166@126.com

**Figure S1.** The feature based molecular networking of *C. salicifolius* with different parts analyzed by LC-MS/MS in positive ionization mode

**Table S1.** The in-house library of coumarins and cinnamic acids from genus *Chimonanthus*

**Table S2.** The annotated nodes in the FBMN of *C. salicifolius*

**Appendix I**

Source codes in R language for screening and identification of compounds in *C. salicifolius.*

**Figures**


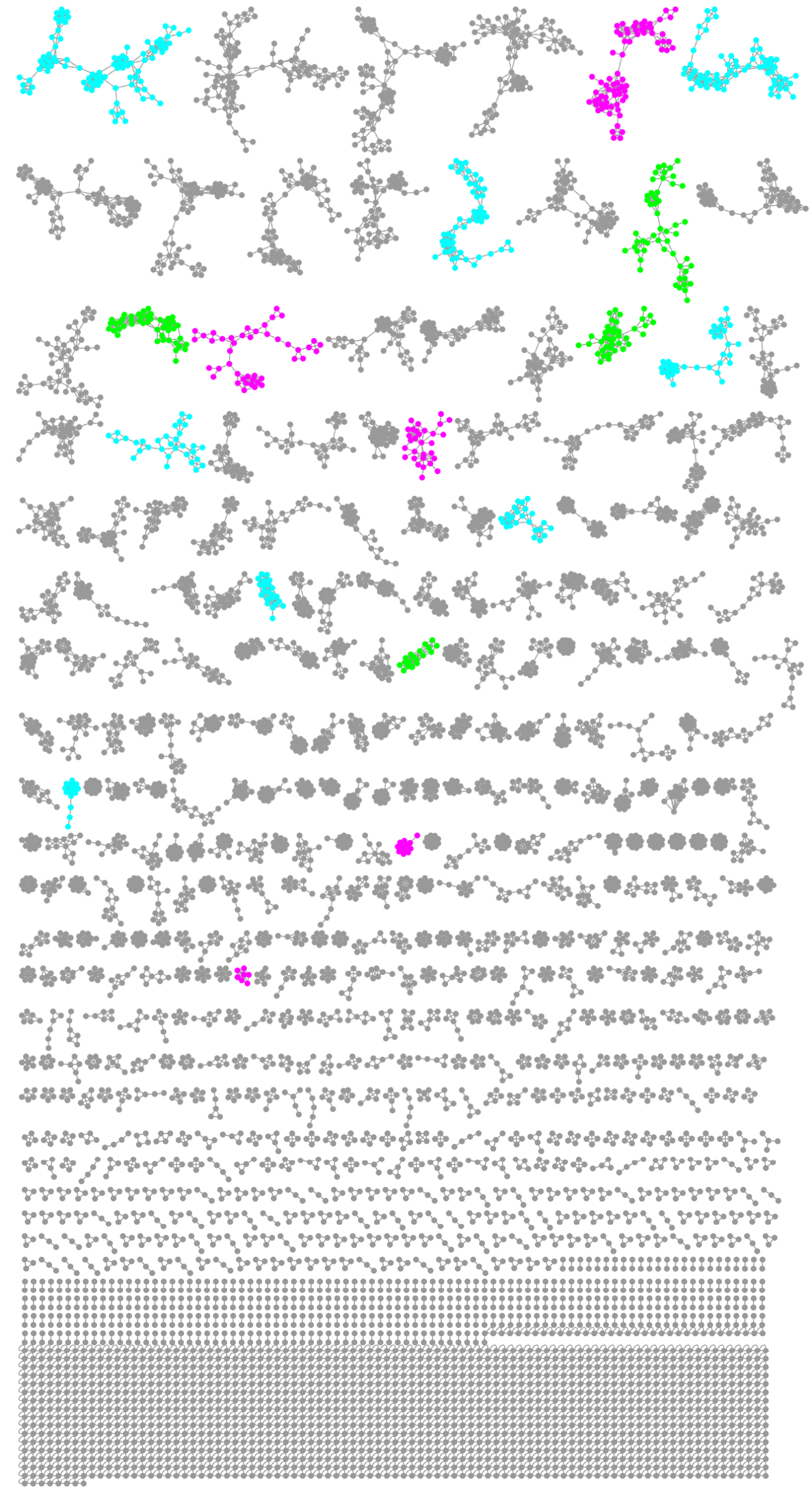


**Figure S1.** The feature based molecular networking of *C. salicifolius* with different parts analyzed by LC-MS/MS in positive ionization mode

**Tables**

**Table S1.** The in-house library of coumarins and cinnamic acids from genus *Chimonanthus*

| **Classification** | **No.** | **Compound name** | **Molecular Formula** | **Origin and Organ** | **Exact Mass (Da)** | **[M+H]^+^ (Da)** | **Sources** |
| --- | --- | --- | --- | --- | --- | --- | --- |
| Coumarins | C1 | scopoletin | C10H8O4 | leaf and root of *C. nitens* Oliv, branch and leaf of *C. salicifolius* | 192.0423 | 193.0501 | References |
| Coumarins | C2 | scopolin | C16H18O9 | root of *C. nitens* Oliv | 354.0951 | 355.1029 | References |
| Coumarins | C3 | fraxetin | C10H8O5 | leaf of *C. praecox* | 208.0372 | 209.0450 | References |
| Coumarins | C4 | isofraxidin | C11H10O5 | leaf of *C. salicifolius* | 222.0528 | 223.0606 | References |
| Coumarins | C5 | calycanthoside | C17H20O10 | root of *C. nitens* Oliv | 384.1056 | 385.1134 | References |
| Coumarins | C6 | 6,7-dimethoxy-coumarin | C11H10O4 | leaf of *C. salicifolius* | 206.0579 | 207.0657 | References |
| Coumarins | C7 | 5,6,7-trimethoxy- coumarin | C12H12O5 | root of *C. nitens* Oliv | 236.0685 | 237.0763 | References |
| Coumarins | C8 | 6,7,8-trimethoxy-coumarin | C12H12O5 | leaf of *C. salicifolius* | 236.0685 | 237.0763 | References |
| Coumarins | C9 | xeroboside | C21H26O13 | leaf of *C. salicifolius* | 486.1373 | 487.1451 | References |
| Coumarins | C10 | nitensoside A | C21H26O14 | root of *C. nitens* Oliv | 502.1323 | 503.1401 | References |
| Coumarins | C11 | nitensoside B | C23H30O15 | root of *C. nitens* Oliv | 546.1585 | 547.1663 | References |
| Coumarins | C12 | hymenain | C20H14O8 | leaf of *C. praecox* | 382.0689 | 383.0767 | References |
| Coumarins | C13 | chimsalicifoliusin A | C21H16O9 | leaf of *C. praecox* | 412.0794 | 413.0872 | References |
| Coumarins | C14 | arteminorin A | C22H18O10 | leaf of *C. praecox* | 442.0900 | 443.0978 | References |
| Coumarins | C15 | 3,3'-biisofraxidin | C22H18O10 | leaf of *C. praecox* | 442.0900 | 443.0978 | References |
| Coumarins | C16 | chimsalicifoliusin B | C20H14O8 | leaf of *C. praecox* | 382.0689 | 383.0767 | References |
| Coumarins | C17 | cleomiscosin B | C20H18O8 | leaf of *C. praecox* | 386.1002 | 387.1080 | References |
| Coumarins | C18 | cleomiscosin A | C20H18O8 | leaf of *C. praecox* | 386.1002 | 387.1080 | References |
| Coumarins | C19 | cleomiscosin C | C21H20O9 | leaf of *C. praecox* | 416.1107 | 417.11185 | References |
| Coumarins | C20 | chimsalicifoliusin C | C31H22O13 | leaf of *C. praecox* | 602.106 | 603.1138 | References |
| Coumarins | C21 | fraxin | C16H18O10 | leaf of *C. praecox* | 370.0900 | 371.0978 | References |
| Coumarins | C22 | euoniside | C17H20O10 | branch and leaf of *C. praecox* | 384.1056 | 385.1134 | References |
| Coumarins | C23 | tomenin | C17H20O10 | root and stem of *C. praecox* | 384.1056 | 385.1134 | References |
| Coumarins | C24 | calycanthoside | C17H20O10 | root and stem of *C. praecox* | 384.1056 | 385.1134 | References |
| Coumarins | C25 | 4, 4′-biisofraxidin | C22H18O10 | stem of *C. salicifolius* | 442.0900 | 443.0978 | References |
| Coumarins | C26 | isofraxoside | C16H18O10 | stem of *C. salicifolius* | 370.0900 | 371.0978 | References |
| Coumarins | C27 | cichoriin | C15H16O9 | stem of *C. salicifolius* | 340.0794 | 341.0872 | References |
| Coumarins | C28 | 6-methoxy-7,8-dihydroxy coumarin-7-*O*-*β*-*D*- apiofuranosyl-(1’’- 6’)-*β*-*D*-glucopyranoside | C21H26O14 | stem of *C. salicifolius* | 502.1323 | 503.1401 | References |
| Coumarins | C29 | 7-hydroxy-6-methoxychroman-2-one | C10H10O4 | root and stem of *C. praecox* | 194.0579 | 195.0657 | References |
| Coumarins | C30 | dimethylfraxetin | C12H12O5 | *C. salicifolius* | 236.0685 | 237.0763 | GNPS |
| Coumarins | C31 | 6-methoxy-7-hydroxycoumarin | C10H8O4 | *C. salicifolius* | 192.0423 | 193.0501 | GNPS |
| Coumarins | C32 | esculetin | C9H6O4 | *C. salicifolius* | 178.0266 | 179.0344 | GNPS |
| Coumarins | C33 | aesculin | C15H16O9 | *C. salicifolius* | 340.0794 | 341.0872 | GNPS |
| Cinnamic acids | CA1 | caffeic acid | C9H8O4 | leaf of *C. nitens* | 180.0423 | 181.0501 | References |
| Cinnamic acids | CA2 | chlorogenic acid | C16H18O9 | leaf of *C. nitens* | 354.0951 | 355.1029 | References |
| Cinnamic acids | CA3 | *p*-coumaroylquinic acid | C16H18O8 | leaf of *C. nitens* | 338.1002 | 339.1080 | References |
| Cinnamic acids | CA4 | *β*-hydroxybenzoyl *p*-coumaric acid anhydribe | C16H12O5 | leaf of *C. nitens* | 284.0685 | 285.0763 | References |
| Cinnamic acids | CA5 | ferulic acid | C10H10O4 | leaf of *C. nitens* Oliv | 194.0579 | 195.0657 | References |
| Cinnamic acids | CA6 | *p*-coumaric acid | C9H8O3 | leaf of *C. nitens* Oliv | 164.0473 | 165.0551 | References |
| Cinnamic acids | CA7 | trans-cinnamic acid | C9H8O2 | flower of *C. praecox* | 148.0524 | 149.0602 | References |
| Cinnamic acids | CA8 | linocinnamarin | C16H20O8 | leaf of *C. praecox* | 340.1158 | 341.1236 | References |
| Cinnamic acids | CA9 | methyl 4-*β*-*D*-glucopyranosyl-ferulate | C17H22O9 | leaf of *C. praecox* | 370.1264 | 371.1342 | References |
| Cinnamic acids | CA10 | methyl *p*-hydroxy-trans-cinnamate | C10H10O3 | leaf of *C. nitens* Oliv | 178.063 | 179.0708 | References |
| Cinnamic acids | CA11 | trans-*p*-hydroxyl ethyl cinnamate | C11H12O3 | leaf of *C. nitens* Oliv | 192.0786 | 193.0864 | References |
| Cinnamic acids | CA12 | cassia cis-trans diphenylpropanoid | C22H22O5 | leaf of *C. nitens* Oliv | 366.1467 | 367.1545 | References |
| Cinnamic acids | CA13 | 3-hydroxy-4-methoxycinnamic acid | C10H10O4 | *C. salicifolius* | 194.0579 | 195.0657 | GNPS |
| Cinnamic acids | CA14 | methoxycinnamic acid | C10H10O3 | *C. salicifolius* | 178.0630 | 179.0708 | GNPS |
| Cinnamic acids | CA15 | neochlorogenic acid | C16H18O9 | *C. salicifolius* | 354.0951 | 355.1029 | GNPS |
| Cinnamic acids | CA16 | 1-feruloyl-*D*-glucose | C16H20O9 | *C. salicifolius* | 356.1107 | 357.1185 | GNPS |

**Table S2.** The annotated nodes in the FBMN of *C. salicifolius*

| Node | Cluster | Precursor mass | MS2 | RT/min | Identification | Adducts | Chemical class | Methods | VIP | auto | part |
| --- | --- | --- | --- | --- | --- | --- | --- | --- | --- | --- | --- |
| 34558 | 375 | 193.0499 | 194.0534 (11.09), 193.05 (100), 178.0264 (21.88), 137.06 (11.88), 133.0288 (42.19), 122.0367 (5.31) | 16.2633 | Scopoletin | [M+H]^+^ | Coumarin | Automatically identified | 0.79572539 | auto | part |
| 16740 | 685 | 193.0499 | 194.0532 (13.33), 193.05 (100), 178.0265 (23.81), 137.06 (12.86), 133.0288 (44.29), 122.0366 (6.67) | 11.5368 | 6-Methoxy-7-hydroxycoumarin | [M+H]^+^ | Coumarin | Automatically identified | 0.38019388 | auto | part |
| 23312 | 31 | 355.1022 | 194.0531 (12), 193.0497 (100), 178.0262 (11), 133.0285 (18) | 7.7963 | Scopolin | [M+H]^+^ | Coumarin | In-house library | 1.21812331 |  |  |
| 8106 | 31 | 425.1951 | 347.2336 (8.08), 210.0489 (11.54), 209.0455 (100), 174.1113 (8.46), 173.1077 (65.38), 144.0811 (6.15), 130.0655 (11.15) | 7.3472 | [Sophoraflavanone G](https://pubchem.ncbi.nlm.nih.gov/compound/72936) | [M+H]^+^ | Flavonoid | Pubchem | 0.22108698 |  |  |
| 24079 | 371 | 1155.2794 | 579.1478 (13.33), 451.1013 (12), 425.0875 (20), 409.0916 (34), 289.0703 (44.67), 287.0547 (38), 271.0606 (41.33), 247.0598 (73.33), 245.0446 (86.67), 229.0504 (17.33), 201.0542 (16.67), 163.0392 (80), 151.0392 (22.67),139.039 (61.33), 135.0442 (93.33), 123.0442 (100) | 9.9246 | Procyanidin tetramer | [M+H]^+^ | Flavonoid | Automatically identified | 0.50337903 | auto | part |
| 32196 | 75 | 779.1998 | 780.2043 (40), 779.2006 (100), 618.1528 (8.67), 617.1463 (31.33), 332.1045 (6), 331.0999 (46), 309.0364 (12.67), 308.0298 (16.67), 185.0428 (7.33), 173.108 (5.6), 85.029 (7.33) | 11.2607 | 3-[4,5-Dihydroxy-6-(hydroxymethyl)-3-[(2*S*,3*R*,4*R*,5*R*,6*S*)-3,4,5-trihydroxy-6-methyloxan-2-yl]oxyoxan-2-yl]oxy-5-hydroxy-2-(4-hydroxyphenyl)-7-[3,4,5-trihydroxy-6-(hydroxymethyl)oxan-2-yl]oxychromen-4-one | [M+Na]^+^ | Flavonoid | Automatically identified | 0.61499193 | auto | part |
| 50686 | 122 | 903.2756 | 449.1081 (25.45), 288.0583 (15.45), 287.055 (100), 129.0547 (5.73), 85.029 (21.82), 71.0498 (15.45) | 10.1547 | 7-[(2*S*,3*R*,4*S*,5*S*,6*R*)-4,5-Dihydroxy-6-(hydroxymethyl)-3-[(2*S*,3*R*,4*R*,5*R*,6*S*)-3,4,5-trihydroxy-6-methyloxan-2-yl]oxyoxan-2-yl]oxy-5-hydroxy-2-(4-hydroxyphenyl)-3-[(2*S*,3*R*,4*S*,5*S*,6*R*)-3,4,5-trihydroxy-6-[[(2*S*,3*R*,4*R*,5*R*,6*S*)-3,4,5-trihydroxy-6-methyloxan-2-yl]oxymethyl]oxan-2-yl]oxychromen-4-one | [M+H]^+^ | Flavonoid | Automatically identified | 0.76138272 | auto | part |
| 50898 | 75 | 779.1997 | 81.1997 (15.5), 780.2053 (50), 779.1967 (100), 729.4429 (12.25), 478.1573 (14.25), 477.1602 (55), 463.9199 (13), 331.1008 (32.5), 324.0245 (18.75), 226.0066 (9), 219.4373 (10), 161.1734 (9), 161.0592 (11.75), 127.5593 (9),119.0494 (9.5), 111.045 (9.25), 85.0292 (42.5), 76.035 (8.75), 71.0498 (27.5), 67.1594 (7.25), 66.5967 (7.5), 60.3512 (7), 54.9005 (7.75) | 15.1642 | [Manghaslin](https://pubchem.ncbi.nlm.nih.gov/compound/11498684) | [M+Na]^+^ | Flavonoid | Automatically identified | 0.78736259 | auto | part |
| 31668 | 122 | 773.2129 | 465.103 (20), 304.0533 (15.88), 303.0499 (100), 287.0551 (5.71), 85.029 (19.41), 71.0498 (14.12) | 9.8129 | 3-[(2*S*,3*R*,4*S*,5*R*,6*S*)-3,4-Dihydroxy-6-methyl-5-[(2*S*,3*R*,4*S*,5*S*,6*R*)-3,4,5-trihydroxy-6-(hydroxymethyl)oxan-2-yl]oxyoxan-2-yl]oxy-2-(3,4-dihydroxyphenyl)-5-hydroxy-7-[3,4,5-trihydroxy-6-(hydroxymethyl)oxan-2-yl]oxychromen-4-one | [M+H]^+^ | Flavonoid | Automatically identified | 0.37629613 | auto | part |
| 50466 | 75 | 763.2046 | 769.3925 (5.19), 765.2142 (7.04), 764.2106 (44.44), 763.2057 (100), 478.1593 (12.96), 477.1583 (77.78), 416.9463 (5.93), 416.7598 (5.19), 413.9633 (5.19), 331.0997 (51.85), 309.0359 (5.93), 308.0293 (15.93), 270.2162 (5.56), 221.3417 (5.19), 187.0578 (8.89), 85.029 (26.3), 71.0499 (21.11) | 16.8744 | Clitorin | [M+Na]^+^ | Flavonoid | Automatically identified | 0.55932278 | auto | part |
| 7209 | 122 | 757.2181 | 450.1115 (5.45), 449.1078 (20.45), 288.0585 (15.68), 287.0551 (100), 85.029 (16.36), 71.0498 (12.05) | 12.4612 | [Kaempferol 3-rutinoside 4'-glucoside](https://pubchem.ncbi.nlm.nih.gov/compound/45359993) | [M+H]^+^ | Flavonoid | Automatically identified | 0.65219678 | auto | part |
| 50436 | 122 | 741.2224 | 288.0582 (15.74), 287.0549 (100), 129.0548 (6.56), 85.0289 (26.23), 71.0498 (19.67) | 17.3598 | [Mauritianin](https://pubchem.ncbi.nlm.nih.gov/compound/5459192) | [M+H]^+^ | Flavonoid | Automatically identified | 0.39947033 | auto | part |
| 4389 | 75 | 633.1419 | 635.1484 (5.43), 634.1483 (34.29), 633.143 (100), 331.1003 (16.29), 324.0232 (8.86) | 17.3211 | Vitamin P | [M+Na]^+^ | Flavonoid | Automatically identified | 0.46233044 | auto | part |
| 50843 | 328 | 697.1598 | 449.1075 (10.37), 288.0582 (16.05), 287.0549 (100), 127.0391 (9.26), 109.0287 (5.06), 85.0289 (10.62), 81.034 (8.15), 69.0341 (5.56) | 13.6026 | 3-[[(2*R*,3*S*,4*S*,5*R*,6*S*)-6-[2-(3,4-Dihydroxyphenyl)-5,7-dihydroxy-4-oxochromen-3-yl]oxy-3,4-dihydroxy-5-[(2*S*,3*R*,4*R*,5*R*,6*S*)-3,4,5-trihydroxy-6-methyloxan-2-yl]oxyoxan-2-yl]methoxy]-3-oxopropanoic acid | [M+H]^+^ | Flavonoid | Pubchem | 0.46732917 |  |  |
| 66461 | 72 | 627.1544 | 465.1018 (6.73), 304.0534 (17.27), 303.0499 (100), 85.029 (10) | 8.9943 | 3-[3,4-Dihydroxy-6-(hydroxymethyl)-5-[3,4,5-trihydroxy-6-(hydroxymethyl)oxan-2-yl]oxyoxan-2-yl]oxy-2-(3,4-dihydroxyphenyl)-5,7-dihydroxychromen-4-one | [M+H]^+^ | Flavonoid | Automatically identified | 0.82927353 | auto | part |
| 28706 | 72 | 449.125 | 288.0643 (16.92), 287.0609 (100), 153.0186 (5.92), 85.0292 (12.31) | 19.4779 | [Scutellaprostin A](https://pubchem.ncbi.nlm.nih.gov/compound/372735) | [M+H]^+^ | Flavonoid | Pubchem | 1.29358740 |  |  |
| 3977 | 75 | 617.147 | 619.154 (5.69), 618.1515 (36.15), 617.1478 (100), 331.1 (15.38), 308.029 (8.46) | 19.9562 | 5,7-Dihydroxy-2-(2-hydroxyphenyl)-3-(((2*R*,3*S*,4*R*,5*R*,6*S*)-3,4,5-trihydroxy-6-((((2*R*,3*S*,4*S*,5*S*,6*S*)-3,4,5-trihydroxy-6-methyltetrahydro-2*H*-pyran-2-yl)oxy)methyl)tetrahydro-2*H*-pyran-2-yl)oxy)-4*H*-chromen-4-one | [M+Na]^+^ | Flavonoid | Automatically identified | 1.18476944 | auto | part |
| 4654 | 122 | 611.1598 | 304.0535 (17.86), 303.05 (100), 85.029 (23.21), 71.0498 (17.86) | 17.331 | Rutin | [M+H]^+^ | Flavonoid | Automatically identified | 1.06748362 | auto | part |
| 50500 | 72 | 611.1594 | 449.1072 (6.53), 288.0585 (18.06), 287.055 (100), 85.029 (7.78) | 10.8134 | [Lilyn](https://pubchem.ncbi.nlm.nih.gov/compound/13647412) | [M+H]^+^ | Flavonoid | Automatically identified | 1.40448531 | auto | part |
| 4290 | 122 | 595.1654 | 288.0583 (20), 287.055 (100), 85.029 (23.64), 71.0498 (17.27) | 20.1608 | Kaempferol-3-*O*-rutinoside | [M+H]^+^ | Flavonoid | Automatically identified | 0.38173272 | auto | part |
| 30631 | 80 | 579.1497 | 427.1029 (5.58), 409.0919 (23.26), 301.0702 (8.6), 287.0549 (25.58), 275.0551 (11.63), 271.0598 (18.6), 257.0444 (12.79), 247.0598 (13.95), 229.0493 (5.7), 203.0336 (8.95), 191.0339 (11.51), 163.039 (39.53), 147.044 (11.63), 139.039 (33.72), 135.0442 (31.4), 127.0392 (100), 123.0443 (89.53) | 10.1972 | Procyanidin B2 | [M+H]^+^ | Flavonoid | Automatically identified | 0.41233405 | auto | part |
| 12637 | 80 | 579.1724 | 427.119 (5.2), 409.1071 (12.6), 301.0784 (10.4), 291.0929 (10.4), 287.0613 (17.4), 271.0649 (13.8), 257.0482 (8), 247.0638 (11.6), 203.0341 (7.2), 191.0338 (8.6), 163.0393 (32), 151.0394 (7), 139.0393 (56), 135.0444 (28), 127.0394 (100), 123.0445 (38) | 7.3386 | [Kaempferitrin](https://pubchem.ncbi.nlm.nih.gov/compound/5486199) | [M+H]^+^ | Flavonoid | Pubchem | 0.43939837 |  |  |
| 23853 | 499 | 577.134 | 425.086 (10), 288.0584 (16.77), 287.055 (100), 163.0393 (6.45), 139.0391 (7.74), 137.0233 (7.74), 127.0392 (11.61), 123.0444 (18.39) | 13.5706 | Procyanidin A2 | [M+H]^+^ | Flavonoid | Automatically identified | 0.47033912 | auto | part |
| 13058 | 499 | 593.1523 | 441.0993 (12.38), 304.0605 (15.24), 303.0572 (100), 287.0623 (5.24), 153.0187 (7.14), 139.0393 (19.05), 123.0444 (27.62) | 9.6058 | [Apigenin 7-[rhamnosyl- (1->2)-galacturonide]](https://pubchem.ncbi.nlm.nih.gov/compound/101428062) | [M+H]^+^ | Flavonoid | Pubchem | 0.45582655 |  |  |
| 30796 | 374 | 573.0845 | 574.1959 (17.61), 574.088 (32.61), 573.1937 (45.65), 573.0845 (100), 529.0962 (16.96), 325.0314 (28.26), 271.0427 (12.83) | 18.3981 | Quercetin 3-*O*-malonylglucoside | [M+Na]^+^ | Flavonoid | Automatically identified | 0.21831794 | auto | part |
| 25617 | 328 | 551.1025 | 304.0533 (17.17), 303.0498 (100), 159.0289 (6.09), 127.0391 (12.83), 109.0287 (10.43), 97.0289 (6.96), 87.0081 (7.39), 85.029 (17.61), 81.034 (11.52), 69.0341 (9.35) | 18.3147 | 3-[[(2*R*,3*S*,4*S*,5*R*,6*S*)-6-[2-(3,4-Dihydroxyphenyl)-5,7-dihydroxy-4-oxochromen-3-yl]oxy-3,4,5-trihydroxyoxan-2-yl]methoxy]-3-oxopropanoic acid | [M+H]^+^ | Flavonoid | Automatically identified | 1.09572727 | auto | part |
| 30875 | 328 | 535.1082 | 288.0587 (18.33), 287.0551 (100), 127.0391 (10.42), 109.0289 (7.5), 87.0082 (5.42), 85.029 (12.92), 81.0341 (9.17), 69.0342 (7.92) | 21.221 | [Luteolin 7-](https://pubchem.ncbi.nlm.nih.gov/compound/5281669)*[O](https://pubchem.ncbi.nlm.nih.gov/compound/5281669)*[-(6''-malonylglucoside)](https://pubchem.ncbi.nlm.nih.gov/compound/5281669) | [M+H]^+^ | Flavonoid | Pubchem | 0.18470448 |  |  |
| 30650 | 247 | 503.1909 | 323.128 (18.75), 311.1281 (17.19), 291.1019 (11.56), 279.1017 (5.94), 231.0807 (9.38), 219.0811 (7.03), 203.0851 (6.41), 199.0755 (12.97), 175.0755 (34.38), 161.0596 (11.09), 138.0632 (10.31), 137.0598 (100), 97.0289 (7.81), 85.029 (20.31), 69.0341 (8.59) | 18.7186 | 2-(Hydroxymethyl)-6-[4-[(2*S*,3*S*)-3-(hydroxymethyl)-5-[(*E*)-3-hydroxyprop-1-enyl]-7-methoxy-2,3-dihydro-1-benzofuran-2-yl]-2-methoxyphenoxy]oxane-3,4,5-triol | [M+H]^+^ | Flavonoid | Automatically identified | 0.70307001 | auto | part |
| 6253 | 50 | 487.0844 | 488.0899 (27.03), 487.2134 (41.89), 487.0851 (100), 325.0307 (11.08), 324.0244 (10.41), 185.0422 (21.62), 114.0919 (7.84) | 16.661 | Isoquercitin | [M+Na]^+^ | Flavonoid | Automatically identified | 0.47461494 | auto | part |
| 66345 | 50 | 283.0809 | 285.1387 (100), 283.0807 (9.41), 269.1072 (20), 257.1074 (38.24), 237.0758 (5.88), 209.0446 (15.88), 191.0701 (13.53), 177.055 (10), 169.0496 (94.12),155.0341 (12.06), 153.0548 (17.35), 141.0547 (35.29), 127.0392 (17.35), 121.0287 (18.82), 105.0703 (23.82), 87.0446 (7.06), 69.0342 (6.47) | 3.999 | [Peniisocoumarin I](https://pubchem.ncbi.nlm.nih.gov/compound/139590018) | [M+H]^+^ | Coumarin | Pubchem | 0.85033461 |  |  |
| 23432 | 50 | 383.0937 | 384.0979 (18.84), 383.0943 (100), 221.0419 (12.32), 185.0422 (20.29) | 5.3372 | [3-(1,3-Benzodioxol-5-yl)-5-phenyl-7](https://pubchem.ncbi.nlm.nih.gov/compound/1765149)*[H](https://pubchem.ncbi.nlm.nih.gov/compound/1765149)*[-furo[3,2-g]chromen-7-one](https://pubchem.ncbi.nlm.nih.gov/compound/1765149) | [M+H]^+^ | Coumarin | Pubchem | 0.79982974 |  |  |
| 4489 | 50 | 471.0893 | 472.0938 (24.44), 471.0899 (100), 309.0368 (17.78), 308.028 (7.78), 185.0424 (9.56) | 19.7533 | [3,3',5,7-Tetraacetoxy-4'-hydroxyflavone](https://pubchem.ncbi.nlm.nih.gov/compound/13714499) | [M+H]^+^ | Flavonoid | Pubchem | 0.60932711 |  |  |
| 25086 | 50 | 485.1061 | 485.1047 (100), 211.0603 (74.49), 193.0496 (47.96), 185.0451 (23.47), 169.0495 (29.59), 147.0436 (5.82), 139.0393 (8.78), 133.0285 (32.65), 115.0393 (12.24),85.0289 (11.22), 69.034 (5.31) | 5.5612 | [3-](https://pubchem.ncbi.nlm.nih.gov/compound/10005544)*[O](https://pubchem.ncbi.nlm.nih.gov/compound/10005544)*[-Methylquercetin tetraacetate](https://pubchem.ncbi.nlm.nih.gov/compound/10005544) | [M+H]^+^ | Flavonoid | Pubchem | 0.33395433 |  |  |
| 24730 | 50 | 323.0748 | 323.0737 (100), 203.0524 (21.54), 185.0421 (73.08), 161.0208 (46.15), 97.0288 (7.69), 85.0291 (24.23), 81.034 (7.69), 69.0342 (11.92) | 6.8851 | [Leucodelphinidin](https://pubchem.ncbi.nlm.nih.gov/compound/44563331) | [M+H]^+^ | Flavonoid | Pubchem | 0.87753548 |  |  |
| 30797 | 24 | 479.1882 | 477.3315 (7.5), 184.0733 (5.75), 169.0497 (5.83), 145.05 (8.08), 133.0497 (100), 115.0392 (76.67), 97.0289 (51.67), 87.0446 (57.5), 85.0289 (54.17), 69.0341 (63.33), 57.0342 (24.17) | 8.1874 | [Thalictricoside](https://pubchem.ncbi.nlm.nih.gov/compound/45359754) | [M+NH_4_]^+^ | Alkaloid | Automatically identified | 0.38013073 | auto | part |
| 51148 | -1 | 466.2651 | 464.3333 (9.71), 455.328 (5.88), 137.1326 (18.82), 133.0497 (17.94), 115.0393 (17.35), 114.0917 (32.35), 97.0289 (12.94), 96.0813 (6.47), 95.0861 (21.18), 85.0291 (16.18), 81.0705 (100), 79.0549 (8.24), 69.0342 (14.12) | 29.5187 | Acuminoside | [M+NH_4_]^+^ | others | Automatically identified | 0.96935010 | auto | part |
| 46696 | 78 | 465.122 | 304.0605 (17.54), 303.0572 (100), 153.0186 (6.14), 85.0292 (14.91) | 16.2434 | Quercetin 7-*O*-glucopyranoside | [M+H]^+^ | Flavonoid | Automatically identified | 1.23874261 | auto | part |
| 31325 | 174 | 465.1029 | 304.0533 (19.09), 303.0499 (100), 229.0497 (5.55), 153.0183 (5.64) | 15.3155 | Spiraeoside | [M+H]^+^ | Flavonoid | Automatically identified | 0.78563099 | auto | part |
| 6604 | 78 | 465.1029 | 304.0533 (18.33), 303.05 (100), 97.0288 (5.37), 85.029 (15.37) | 16.6828 | [Isoquercetin](https://pubchem.ncbi.nlm.nih.gov/compound/5280804) | [M+H]^+^ | Flavonoid | Automatically identified | 0.68190526 | auto | part |
| 50988 | 829 | 465.1023 | 467.1061 (6.57), 304.0532 (18.57), 303.05 (100), 147.0441 (10.71) | 10.2482 | [Hyperoside](https://pubchem.ncbi.nlm.nih.gov/compound/5281643) | [M+H]^+^ | Flavonoid | Automatically identified | 0.61013713 | auto | part |
| 22586 | 31 | 463.1409 | 302.0817 (16.86), 301.078 (100), 287.0564 (5.43), 286.0533 (31.43), 258.057 (12.86) | 20.4228 | Peonidin 3-*O*-galactoside | [M+H]^+^ | Flavonoid | Automatically identified | 0.26342995 | auto | part |
| 23312 | 31 | 355.1022 | 194.0531 (12), 193.0497 (100), 178.0262 (11), 133.0285 (18) | 7.7963 | [Neochlorogenic acid](https://pubchem.ncbi.nlm.nih.gov/compound/5280633) | [M+H]^+^ | Cinnamic acid | In-house library | 1.14197465 |  |  |
| 13069 | 31 | 457.113 | 209.0453 (100), 181.0503 (6.55), 163.0395 (6.9), 149.0236 (8.28), 85.029 (5.17) | 10.2493 | [Epicatechin 3-](https://pubchem.ncbi.nlm.nih.gov/compound/467296)*[O](https://pubchem.ncbi.nlm.nih.gov/compound/467296)*[-(3-](https://pubchem.ncbi.nlm.nih.gov/compound/467296)*[O](https://pubchem.ncbi.nlm.nih.gov/compound/467296)*[-methylgallate)](https://pubchem.ncbi.nlm.nih.gov/compound/467296) | [M+H]^+^ | Flavonoid | Pubchem | 0.45533122 |  |  |
| 17065 | 160 | 451.1407 | 451.1415 (5.5), 291.094 (5.43), 289.0766 (33.57), 205.0509 (7.86), 181.0499 (5.36), 163.039 (25), 147.0445 (34.29), 139.0392 (100), 123.0445 (35.71), 119.0498 (11.43), 97.0291 (8.57), 85.0292 (22.14), 69.0345 (12.86) | 5.2174 | 5-Hydroxy-3-(3-hydroxy-4,5-dimethoxyphenyl)-6-methoxy-7-phenylmethoxychromen-4-one | [M+H]^+^ | Flavonoid | Pubchem | 0.33021940 |  |  |
| 23417 | 160 | 453.1374 | 291.0863 (26.79), 165.0548 (13.57), 163.0392 (6.07), 147.0442 (17.5), 140.0423 (8.93), 139.0391 (100), 123.0443 (57.14), 85.029 (10.71) | 7.1029 | (2*R*,3*R*,4*S*,5*S*,6*R*)-2-[[2-(3,4-dihydroxyphenyl)-5,7-dihydroxy-3,4-dihydro-2*H*-chromen-3-yl]oxy]-6-(hydroxymethyl)oxane-3,4,5-triol | [M+H]^+^ | Flavonoid | Automatically identified | 0.60699389 | auto | part |
| 30826 | 24 | 450.1956 | 315.1043 (5.36), 247.0959 (7.83), 235.0971 (8.99), 217.0863 (15.94), 205.087 (8.41), 187.0755 (17.39), 175.0758 (8.99), 163.0759 (14.35), 145.0646 (34.78), 133.0497 (100), 115.0393 (98.55), 97.0289 (79.71), 85.0289 (79.71), 69.0341 (81.16), 57.0343 (30.43) | 7.8678 | Osmanthuside H | [M+NH_4_]^+^ | Benzene derivative | Automatically identified | 1.01694434 | auto | part |
| 28252 | 174 | 449.1076 | 288.0584 (18.06), 287.055 (100) | 11.0923 | Kaempferol-3-*O*-glucoside | [M+H]^+^ | Flavonoid | Automatically identified | 1.37690604 | auto | part |
| 30755 | 174 | 449.1075 | 288.0584 (16.67), 287.0549 (100), 153.0181 (5.19) | 18.599 | [Kaempferol-3-](https://pubchem.ncbi.nlm.nih.gov/compound/5282149)*[O](https://pubchem.ncbi.nlm.nih.gov/compound/5282149)*[-galactoside](https://pubchem.ncbi.nlm.nih.gov/compound/5282149) | [M+H]^+^ | Flavonoid | Automatically identified | 0.24977723 | auto | part |
| 25440 | 72 | 449.1072 | 288.0583 (17.65), 287.055 (100), 153.0182 (5.53), 85.029 (14.12) | 20.0698 | Astragalin | [M+H]^+^ | Flavonoid | Automatically identified | 1.33588037 | auto | part |
| 6965 | 24 | 448.2169 | 223.0603 (6.55), 147.0658 (22.62), 129.0547 (38.1), 85.0289 (100), 83.05 (6.19), 75.0449 (5.48), 71.0498 (67.86), 57.0342 (11.55) | 15.528 | 2-Phenylethyl 6-*O*-(6-deoxy- alpha-*L*-mannopyranosyl)-beta-*D*-glucopyranoside | [M+NH_4_]^+^ | Benzene derivative | Automatically identified | 0.45689009 | auto | part |
| 6844 | 68 | 435.0906 | 304.0533 (18.84), 303.05 (100), 229.0501 (5.22), 191.0339 (6.96), 153.0181 (5.07), 73.029 (21.74) | 18.2199 | Quercetin-3-arabinoside | [M+H]^+^ | Flavonoid | Automatically identified | 1.26351435 | auto | part |
| 66310 | 68 | 403.1015 | 403.0995 (100), 289.0705 (10.69), 271.0604 (26.9), 241.0472 (11.72), 223.0961 (9.66), 195.1011 (7.24), 181.0495 (12.07), 163.0388 (12.41), 151.0395 (11.38), 135.0443 (19.31), 127.0391 (14.83), 123.0443 (27.93), 86.097 (12.07), 85.029 (8.28), 69.0341 (7.93) | 14.4802 | [8-](https://www.ncbi.nlm.nih.gov/pcsubstance/?term="8-C-alpha-L-Arabinopyranosylapigenin"[CompleteSynonym] AND 118707634[StandardizedCID]" \o "" \t "https://pubchem.ncbi.nlm.nih.gov/compound/_blank)*[C](https://www.ncbi.nlm.nih.gov/pcsubstance/?term="8-C-alpha-L-Arabinopyranosylapigenin"[CompleteSynonym] AND 118707634[StandardizedCID]" \o "" \t "https://pubchem.ncbi.nlm.nih.gov/compound/_blank)*[-alpha-](https://www.ncbi.nlm.nih.gov/pcsubstance/?term="8-C-alpha-L-Arabinopyranosylapigenin"[CompleteSynonym] AND 118707634[StandardizedCID]" \o "" \t "https://pubchem.ncbi.nlm.nih.gov/compound/_blank)*[L](https://www.ncbi.nlm.nih.gov/pcsubstance/?term="8-C-alpha-L-Arabinopyranosylapigenin"[CompleteSynonym] AND 118707634[StandardizedCID]" \o "" \t "https://pubchem.ncbi.nlm.nih.gov/compound/_blank)*[-Arabinopyranosylapigenin](https://www.ncbi.nlm.nih.gov/pcsubstance/?term="8-C-alpha-L-Arabinopyranosylapigenin"[CompleteSynonym] AND 118707634[StandardizedCID]" \o "" \t "https://pubchem.ncbi.nlm.nih.gov/compound/_blank) | [M+H]^+^ | Flavonoid | Pubchem | 1.21548451 |  |  |
| 66195 | 68 | 405.1164 | 406.1186 (24.14), 405.1154 (100), 233.0806 (6.55), 181.0497 (9.48), 167.0703 (13.79), 137.0597 (10.86) | 15.1206 | [5,3'-Dihydroxy-3,6,7,8,4'-pentamethoxyflavone](https://pubchem.ncbi.nlm.nih.gov/compound/369954) | [M+H]^+^ | Flavonoid | Pubchem | 0.83385066 |  |  |
| 23738 | 24 | 434.2009 | 376.1637 (6.06), 285.1337 (5.64), 173.1074 (11.7), 150.0763 (10.64), 145.0495 (27.66), 133.0497 (100), 127.0393 (13.83), 115.0393 (82.98), 97.0289 (59.57), 87.0446 (48.94), 85.0289 (84.04), 69.0341 (69.15), 57.0343 (23.4) | 13.1671 | [Sayaendoside](https://pubchem.ncbi.nlm.nih.gov/compound/10993414) | [M+NH_4_]^+^ | Benzene derivative | Automatically identified | 0.20896672 | auto | part |
| 31728 | 1081 | 433.113 | 287.0551 (100), 181.0494 (10.5), 167.0706 (7), 129.055 (7.5), 85.029 (45), 71.0498 (41.5) | 22.6789 | Afzelin | [M+H]^+^ | Flavonoid | Automatically identified | 1.23287604 | auto | part |
| 26218 | 24 | 420.1872 | 419.1311 (16.84), 260.0894 (24.21), 173.1073 (21.05), 145.0497 (13.16), 133.0497 (35.26), 115.0393 (43.16), 97.0289 (50), 91.0547 (100), 87.0446 (15.26), 85.0289 (48.42), 73.029 (46.84), 69.0342 (28.95), 61.0292 (8.95), 57.0343 (16.84) | 10.2465 | [Icariside F2](https://pubchem.ncbi.nlm.nih.gov/compound/14079045) | [M+NH_4_]^+^ | Benzene derivative | Automatically identified | 0.44540290 | auto | part |
| 32486 | 68 | 419.0968 | 287.0551 (100), 259.0599 (11.52), 181.0497 (12.12), 167.0704 (27.27), 73.0291 (19.7) | 20.0782 | Kaempferol 3-*O*-arabinoside | [M+H]^+^ | Flavonoid | Automatically identified | 0.20537809 | auto | part |
| 6644 | -1 | 418.1965 | 409.1841 (5.88), 233.0418 (7.65), 216.0349 (11.47), 215.0316 (100), 193.05 (12.65) | 11.9719 | [4-Acetyloxy-2,5-dihydroxy-6-(hydroxymethyl)oxan-3-yl] (*E*)-3-(4-hydroxyphenyl)prop-2-enoate | [M+K]^+^ | Cinnamic acid | Automatically identified | 0.93937081 | auto | part |
| 50985 | 233 | 407.0745 | 418.1973 (51.52), 303.0861 (9.09), 257.128 (8.48), 237.0756 (7.88), 219.0649 (7.27), 208.097 (5.15), 194.0813 (57.58), 151.0388 (7.27), 147.0435 (6.06), 137.0234 (100), 123.0444 (7.58), 85.0288 (5.15), 72.0815 (10) | 5.8849 | Pistillarin | [M+H]^+^ | Alkaloid | Automatically identified | 0.60134351 | auto | part |
| 66322 | 208 | 407.2389 | 409.1813 (100), 407.0935 (77.78), 389.2283 (12.78), 371.2178 (23.06), 353.2062 (12.22), 323.1966 (63.89), 245.0419 (30.56), 244.058 (21.67), 230.1385 (20.28), 196.1335 (8.33), 164.1071 (20.83), 96.0447 (13.61), 86.097 (44.44), 85.0289 (9.44), 69.0706 (12.78) | 10.1668 | FruLeuIle | [M+H]^+^ | Alkaloid | Automatically identified | 0.91819538 | auto | part |
| 25862 | 935 | 405.1906 | 193.0862 (18.2), 168.0739 (10.2), 167.0704 (100), 163.0756 (13.8), 161.0598 (6.8), 131.0493 (8), 123.0443 (8.6), 107.0495 (7.4), | 15.7965 | [Fragransin B2](https://pubchem.ncbi.nlm.nih.gov/compound/13870574) | [M+H]^+^ | Benzene derivative | Automatically identified | 0.83883905 | auto | part |
| 5593 | 604 | 384.1141 | 384.1148 (25.49), 340.2594 (8.82), 253.0753 (11.18), 252.0728 (100), 206.0677 (6.67), 192.0516 (16.47), 188.0567 (11.18), 162.0774 (33.33), 148.0619 (7.06), 136.0618 (17.84) | 4.4065 | Succinoadenosine | [M+H]^+^ | Alkaloid | Automatically identified | 0.75072057 | auto | part |
| 24072 | 34 | 381.1148 | 381.1158 (38.16), 220.0685 (14.47), 219.0653 (100), 173.0599 (18.42), 147.0443 (7.11), 145.065 (10), 131.0495 (5.53), 85.0289 (6.32) | 7.5778 | 3-[4-Methoxy-2-[(2*S*,3*R*,4*S*,5*S*,6*R*)-3,4,5-trihydroxy-6-(hydroxymethyl)oxan-2-yl]oxyphenyl]propanoic acid | [M+Na]^+^ | Cinnamic acid | Automatically identified | 0.81729254 | auto | part |
| 66275 | 34 | 357.1181 | 357.1887 (100), 207.0652 (28), 195.0654 (92), 193.05 (32.4), 165.091 (31.6), 153.0547 (16.8), 145.0497 (23.2), 137.096 (37.6), 127.0392 (28), 109.1016 (28), 97.0288 (34.4), 85.029 (80), 81.0341 (25.6), 69.0342 (76) | 18.2874 | [1-Feruloyl-](https://pubchem.ncbi.nlm.nih.gov/compound/13962928)*[D](https://pubchem.ncbi.nlm.nih.gov/compound/13962928)*[-glucose](https://pubchem.ncbi.nlm.nih.gov/compound/13962928) | [M+H]^+^ | Cinnamic acid | In-house library | 1.32871895 |  |  |
| 34050 | 34 | 385.1123 | 385.1054 (8.18), 383.1321 (5.96), 223.0599 (31.31), 205.0496 (100), 193.0497 (5.96), 149.0599 (5.15), 121.0287 (9.39), 85.0288 (5.86) | 6.7415 | [6,8-Dimethoxy-7-[3,4,5-trihydroxy-6-(hydroxymethyl)oxan-2-yl]oxychromen-2-one](https://pubchem.ncbi.nlm.nih.gov/compound/12302276) | [M+H]^+^ | Cinnamic acid | Pubchem | 0.60555275 |  |  |
| 66489 | 34 | 397.1099 | 399.1279 (15.26), 397.1104 (36.84), 237.0396 (20.26), 235.06 (97.37), 219.029 (100), 191.0703 (12.89), 189.0548 (17.89),163.039 (13.16), 147.0441 (16.32), 127.0394 (5.26), 97.0288 (5.26), 85.029 (11.84), 69.0341 (10.53) | 5.9342 | [2-[(*E*)-3-Oxo-3-phenylprop-1-enyl]phenyl] 2- oxochromene-3-carboxylate | [M+H]^+^ | Coumarin | Pubchem | 1.40674382 |  |  |
| 66232 | 34 | 399.0909 | 399.1265 (5.62), 397.1093 (5.69), 299.0729 (5.77), 238.1431 (5.54), 237.0394 (21.54), 220.0322 (13.85), 219.0289 (100) | 6.3336 | *[O](https://pubchem.ncbi.nlm.nih.gov/compound/46926117)*[-Sinapoylglucarolactone](https://pubchem.ncbi.nlm.nih.gov/compound/46926117) | [M+H]^+^ | Cinnamic acid | Pubchem | 1.22840160 |  |  |
| 1398 | 34 | 441.1358 | 280.0898 (17.22), 279.0864 (100), 233.0811 (11.11) | 16.5788 | [3,3'-[(4-Ethylphenyl)methanediyl]bis(4-hydroxy-2](https://pubchem.ncbi.nlm.nih.gov/compound/54679216)*[H](https://pubchem.ncbi.nlm.nih.gov/compound/54679216)*[-chromen-2-one)](https://pubchem.ncbi.nlm.nih.gov/compound/54679216) | [M+H]^+^ | Coumarin | Pubchem | 1.19647407 |  |  |
| 5717 | 34 | 457.1269 | 458.2463 (25), 457.2413 (100), 455.2251 (27.5), 455.1531 (8.61), 437.3401 (8.06), 411.169 (17.78), 279.1932 (6.94), 277.1775 (10.28), 237.0285 (6.94), 115.0952 (6.67), 114.0917 (19.17), 96.0813 (6.94), 86.0971 (5.28) | 21.9585 | 3-[(4-Ethoxyphenyl)-(4-hydroxy-2-oxochromen-3-yl)methyl]-4-hydroxychromen-2-one | [M+H]^+^ | Coumarin | Pubchem | 1.14397178 |  |  |
| 50833 | 217 | 375.2386 | 376.2194 (7.06),375.1995 (7.65), 213.1849 (57.06), 201.1133 (14.71), 195.1745 (40), 177.1638 (100), 165.0911 (12.35), 153.1277 (11.18), 145.0498 (18.24), 137.0962 (28.24), 135.1168 (30), 127.0391 (24.12), 121.1013 (42.94),109.1014 (50.59), 107.0859 (32.35), 99.0809 (26.47), 97.0289 (30), 95.086 (55.88), 93.0704 (22.35), 85.0289 (88.24), 81.0705 (40), 69.0705 (44.12), 67.0549 (20), 55.055 (22.94) | 20.5473 | 4-(4-Hydroxy-2,6,6-trimethyl-1-cyclohexen-1-yl)-2-butanyl beta-*D*-glucopyranoside | [M+H]^+^ | others | Automatically identified | 0.63974127 | auto | part |
| 32059 | 217 | 373.2236 | 211.1693 (67.8), 193.1588 (69.49), 175.1482 (37.29),151.1488 (8.47), 135.1169 (100), 119.0856 (32.2), 109.1015 (96.61), (23.73), 95.086 (33.9), 85.0289 (16.95), 81.0704 (16.95), 69.0705 (14.75), 67.0547 (15.59) | 20.8064 | (2,6,6-Trimethyl-4-oxo-2-cyclohexen-1-yl)-2-butanyl beta-D-glucopyranoside | [M+H]^+^ | others | Automatically identified | 1.24349187 | auto | part |
| 30608 | 31 | 371.0972 | 210.048 (11.9), 209.0446 (100), 194.0211 (25.24), 149.0234 (10.48) | 6.316 | [Isofraxoside](https://pubchem.ncbi.nlm.nih.gov/compound/11508953) | [M+H]^+^ | Coumarin | In-house library |  |  |  |
| 66277 | 240 | 367.1501 | 369.1154 (100), 367.1873 (39.6), 349.1389 (44), 332.1129 (24.8), 276.123 (15.2), 258.1124 (10), 229.0972 (56), 207.0636 (20), 188.0706 (68), 174.0918 (24.8), 168.0807 (15.2), 163.0392 (19.6), 156.0809 (34.4), 146.0602 (68), 144.081 (36.4), 130.0652 (31.2), 118.0655 (23.6), 89.0602 (16), 85.0289 (16.8), 69.0342 (18.4) | 4.9388 | 1-beta-*D*-Glucopyranosyl-*L*-tryptophan | [M+H]^+^ | Alkaloid | Automatically identified | 1.33392623 | auto | part |
| 23357 | 285 | 355.1024 | 194.0531 (12), 193.0497 (100), 85.0289 (10) | 8.9071 | Undulatoside A | [M+H]^+^ | Coumarin | Automatically identified | 0.51002810 | auto | part |
| 23373 | -1 | 347.2229 | 347.2228 (10.48), 316.1809 (11.43), 174.1108 (12.38), 173.1074 (100), 158.0963 (18.57), 144.0809 (11.9), 130.0652 (25.71) | 7.172 | Calycanthine | [M+H]^+^ | Alkaloid | Automatically identified | 0.89376212 | auto | part |
| 23329 | 19 | 347.2229 | 347.2233 (8.2), 316.1809 (9.4), 174.111 (13), 173.1075 (100), 144.0809 (26), 130.0653 (24) | 11.3877 | [(-)-Chimonanthine](https://pubchem.ncbi.nlm.nih.gov/compound/599086) | [M+H]^+^ | Alkaloid | Automatically identified |  | auto | part |
| 30614 | 286 | 341.0866 | 180.0373 (11.46), 179.034 (100), 133.0285 (9.17), 123.0442 (9.17) | 5.6028 | Aesculin | [M+H]^+^ | Coumarin | In-house library | 0.34743426 |  |  |
| 23601 | 1063 | 330.1697 | 331.1728 (14.64), 330.1698 (50), 193.1054 (14.64), 192.102 (100), 175.0757 (11.79), 143.0492 (16.79), 137.0598 (39.29) | 11.0962 | Reticuline | [M+H]^+^ | Alkaloid | Automatically identified | 1.50953645 | auto | part |
| 23498 | 1143 | 328.1548 | 329.1581 (19.5), 328.1543 (100), 312.1214 (8.5), 298.1152 (16.5), 297.1122 (70), 282.0885 (24.5), 265.0859 (60), 237.0911 (17), 222.0676 (13), 207.0808 (9), 191.0856 (13), 137.06 (7) | 9.7447 | Isoboldine | [M+H]^+^ | Alkaloid | Automatically identified | 0.71264628 | auto | part |
| 24538 | 238 | 328.1384 | 311.132 (19.5), 310.1286 (100), 292.1181 (46.5), 265.1271 (5.5), 264.1235 (36), 246.1123 (8.5), 244.0973 (6), 178.0866 (16.5), 166.0864 (27), 144.081 (10), 132.0809 (50), 120.0811 (65), 105.0705 (13.5), 97.0289 (15), 91.0547 (17.5), 87.0321 (11.5), 85.029 (10.5), 69.0342 (10.5) | 3.3297 | *N*-(1-Deoxy-1-fructosyl)phenylalanine | [M+H]^+^ | Alkaloid | Automatically identified | 1.37880201 | auto | part |
| 23914 | 60 | 327.1588 | 327.1574 (9.72), 295.1324 (10.83), 163.0754 (52.78), 151.0755 (11.11), 137.0598 (100), 135.0805 (13.06), 133.0649 (36.11), 131.0494 (20), 121.0651 (8.61), 114.0916 (63.89), 105.0703 (10.56), 103.0546 (18.89) | 19.3121 | 2,3-Bis[(4-hydroxy-3-methoxyphenyl)methyl]butane-1,4-diol | [M-2H_2_O+H]^+^ | Benzene derivative | Automatically identified | 0.76793142 | auto | part |
| 3307 | 378 | 324.1796 | 324.1786 (21.74), 268.1124 (56.52), 256.1113 (100), 197.0709 (7.68), 185.0711 (23.19), 169.076 (15.94), 157.0762 (23.19), 156.0685 (12.46), 154.1227 (14.49), 130.0655 (8.99), 107.0859 (6.09), 105.0705 (6.09), 93.0707 (6.38), 81.0706 (10.14), 69.0707 (71.01), 55.0551 (6.09) | 25.9019 | Neoechinulin A | [M+H]^+^ | Alkaloid | Automatically identified | 0.57996502 | auto | part |
| 13206 | 664 | 319.0539 | 319.0532 (100), 301.1242 (7.27), 273.0437 (54.55), 257.1104 (6.36), 245.0478 (11.36), 217.0506 (7.73), 177.0545 (5.91), 165.0182 (15), 153.0182 (33.18), 147.0442 (10), 137.0232 (7.73), 111.0081 (8.64), 81.0706 (5.91) | 13.6605 | Myricetin | [M+H]^+^ | Flavonoid | Automatically identified | 0.52034414 | auto | part |
| 12947 | 343 | 317.0746 | 318.0783 (21.61), 317.0742 (100), 302.0493 (20), 285.0451 (7.74), 229.0524 (6.77), 165.0551 (13.87), 153.0185 (19.68), 123.0445 (6.77) | 19.1404 | Isorhamnetin | [M+H]^+^ | Flavonoid | Automatically identified | 0.22888964 | auto | part |
| 33073 | 117 | 309.0961 | 148.0475 (11.25), 147.0441 (100), 119.0494 (23.12), 91.0548 (7.5), 69.0342 (7.19) | 16.0435 | beta-*D*-Glucopyranose, 1-*O*-[(2*E*)-3-(2-hydroxyphenyl)-1-oxo-2-propen-1-yl]- | [M-H_2_O+H]^+^ | Cinnamic acid | Automatically identified | 0.45016199 | auto | part |
| 30852 | 117 | 163.0756 | 165.0548 (55.56), 163.0754 (45), 147.0442 (52.78), 135.0442 (20), 131.0493 (100), 119.0495 (26.11), 103.0547 (72.22), 95.0497 (6.11), 91.0547 (18.89), 89.0391 (11.67), 79.0548 (7.22) | 10.0798 | [Methyl cinnamate](https://pubchem.ncbi.nlm.nih.gov/compound/637520) | [M+H]^+^ | Cinnamic acid | pubchem | 0.98801426 |  |  |
| 12643 | 530 | 307.0889 | 163.0392 (8.64), 140.0426 (8.27), 139.0392 (100) | 5.4584 | Epigallocatechin | [M+H]^+^ | Flavonoid | Automatically identified | 0.93497472 | auto | part |
| 50606 | 544 | 305.065 | 305.0658 (11.58), 287.0548 (17.37), 259.0602 (47.37), 243.1692 (5.79), 231.0652 (34.21), 217.0494 (7.89), 193.0496 (28.95), 171.0289 (22.89), 153.0183 (100), 149.0234 (52.63), 127.0391 (17.11), 123.0443 (65.79), 84.0814 (13.16), 67.0185 (7.63) | 6.2508 | Taxifolin | [M+H]^+^ | Flavonoid | Automatically identified | 1.19948151 | auto | part |
| 36962 | 154 | 303.094 | 303.0937 (45.35), 179.0343 (10), 177.055 (45.35), 163.0394 (8.26), 153.0186 (100), 117.034 (8.14), 89.0393 (7.09), 67.0187 (6.28) | 18.1498 | Hesperitin | [M+H]^+^ | Flavonoid | Automatically identified | 0.33639186 | auto | part |
| 16746 | 680 | 303.0573 | 304.0606 (20), 303.0572 (100), 229.0525 (10), 153.0186 (12.67), 137.0239 (9.33) | 16.7219 | Quercetin | [M+H]^+^ | Flavonoid | Automatically identified | 1.29748433 | auto | part |
| 21785 | 214 | 301.0779 | 302.0817 (22.22), 301.0782 (100), 287.0563 (8.89), 286.0532 (50), 259.0603 (5.56), 258.057 (34.72), 167.0343 (13.61), 153.0186 (5.83), 149.0602 (7.08), 107.0498 (5.69), 70.066 (5.83) | 20.4506 | Diosmetin | [M+H]^+^ | Flavonoid | Automatically identified | 0.26821084 | auto | part |
| 66191 | -1 | 298.0977 | 136.0619 (100), 61.0114 (7.5) | 4.7822 | [5'-Methylthioadenosine](https://pubchem.ncbi.nlm.nih.gov/compound/439176) | [M+H]^+^ | Alkaloid | Automatically identified | 1.28748176 | auto | part |
| 16919 | 22 | 295.2344 | 295.2334 (24.39), 277.2218 (53.66), 259.2106 (11.1), 241.1984 (6.83), 219.1771 (8.9), 185.1333 (6.83), 173.133 (10.12), 151.112 (21.95), 135.1172 (23.17), 133.1016 (21.95), 121.1016 (45.12), 109.1018 (34.15), 107.0861 (46.34), 105.0705 (39.02), 95.0862 (84.15), 93.0706 (69.51), 91.055 (37.8), 83.0864 (30.49), 81.0707 (100), 79.0551 (51.22),71.0499 (34.15), 69.0708 (46.34), 67.0551 (85.37), 55.0552 (52.44) | 30.8587 | 13-Keto-9*Z*,11*E*-octadecadienoic acid | [M+H]^+^ | Fatty acid | Automatically identified | 1.29204667 | auto | part |
| 37312 | 425 | 295.1727 | 295.1719 (5.12), 137.0793 (9.77), 136.076 (100), 119.0496 (16.05), 91.0549 (23.26), 86.0972 (7.67) | 8.5228 | Tyr-Ile | [M+H]^+^ | Alkaloid | Automatically identified | 0.41997965 | auto | part |
| 37146 | 425 | 295.1724 | 166.0867 (12.96), 137.0795 (9.01), 136.076 (100), 120.0813 (28.17), 119.0496 (15.49), 91.055 (23.94), 86.0972 (10.56), 84.0452 (9.72) | 6.9133 | Tyr-Leu | [M+H]^+^ | Alkaloid | Automatically identified | 0.30612266 | auto | part |
| 11846 | 425 | 295.1359 | 148.0608 (8), 121.0845 (9.56), 120.0813 (100), 86.0972 (10.22), 84.0452 (7.56) | 3.4006 | Glutamylphenylalanine | [M+H]^+^ | Alkaloid | Automatically identified | 0.39916091 | auto | part |
| 51111 | 425 | 295.1337 | 148.0608 (8), 121.0845 (9.56), 120.0813 (100), 86.0972 (10.22), 84.0452 (7.56) | 3.3203 | Glu-Phe | [M+H]^+^ | Alkaloid | Automatically identified | 0.29419704 | auto | part |
| 41941 | 1025 | 293.2179 | 293.2175 (20.69), 275.2064 (31.03), 257.1939 (5.69), 165.1276 (5.52), 147.1171 (12.93), 133.1015 (13.79), 119.086 (22.41), 109.0654 (20.69), 105.0705 (29.31), 95.0862 (32.76), 93.0706 (27.59), 91.0549 (24.14), 81.0707 (100), 79.055 (32.76), 67.0551 (36.21), 55.0552 (22.41) | 29.5134 | 9(*S*)-HpOTrE | [M-H_2_O+H]^+^ | Fatty acid | Automatically identified | 1.23699735 | auto | part |
| 7051 | 25 | 291.0933 | 291.0923 (7.65), 207.0659 (6.47), 165.055 (14.71), 147.0443 (21.18), 140.0426 (8.24), 139.0393 (100), 123.0445 (70.59) | 7.4458 | Catechin | [M+H]^+^ | Flavonoid | Automatically identified | 0.32327552 | auto | part |
| 37016 | 25 | 291.0925 | 291.0923 (6.4), 207.0663 (7.2), 165.055 (15.6), 161.0598 (7.6), 147.0444 (21.2), 139.0392 (100), 123.0445 (72), 119.0497 (6.4) | 5.7385 | (-)-Epicatechin | [M+H]^+^ | Flavonoid | Automatically identified | 0.27408885 | auto | part |
| 41708 | 544 | 289.0768 | 289.0762 (26.67), 287.0605 (46.67), 271.0641 (14.17), 243.0684 (31.67), 215.071 (35), 171.0291 (18.33), 153.0183 (80), 149.0235 (40), 147.0443 (23.33), 139.039 (100), 135.0442 (55), 127.0392 (25), 123.0443 (75), 111.0444 (20), 107.0496 (46.67),91.0549 (13.17), 68.9979 (10.5) | 7.7984 | Dihydrokaempferol | [M+H]^+^ | Flavonoid | Automatically identified | 1.29735490 | auto | part |
| 36989 | 154 | 287.0974 | 288.1003 (9.23), 287.0973 (46.15), 171.029 (14.23), 161.06 (35.77), 154.0219 (8.85), 153.0185 (100), 133.0651 (15.77), 67.0187 (7.31) | 23.7478 | Isosakuranetin | [M+H]^+^ | Flavonoid | Automatically identified | 0.30909793 | auto | part |
| 33719 | 467 | 287.0911 | 287.0551 (100), 245.0811 (43.9), 227.0702 (46.34), 175.0391 (34.15), 153.0184 (10), 135.0441 (5.37), 121.0286 (9.27) | 24.6392 | [Qianhucoumarin C](https://pubchem.ncbi.nlm.nih.gov/compound/196741) | [M-H_2_O+H]^+^ | Coumarin | Automatically identified | 0.88621659 | auto | part |
| 31476 | 467 | 245.0834 | 245.081 (100), 227.0706 (9.26), 217.0865 (8.52), 203.0701 (7.41), 175.0392 (88.89), 147.0442 (13.33), 91.0549 (7.41) | 24.6076 | [Graveolone](https://pubchem.ncbi.nlm.nih.gov/compound/177751) | [M+H]^+^ | Coumarin | Pubchem | 0.61962709 |  |  |
| 19202 | 56 | 287.061 | 288.0645 (18.21), 287.0609 (100), 153.0186 (12.82), 121.029 (7.69) | 19.5895 | Kaempferol | [M+H]^+^ | Flavonoid | Automatically identified | 1.30081426 | auto | part |
| 36986 | 425 | 281.1555 | 281.156 (5.53), 137.0795 (8.95), 136.076 (100), 119.0496 (16.58), 91.055 (22.37), 72.0816 (6.32) | 3.5522 | Tyr-Val | [M+H]^+^ | Alkaloid | Automatically identified | 0.29910371 | auto | part |
| 74 | 22 | 279.2378 | 279.2375 (32.5), 277.184 (6), 149.0236 (15.83), 137.1328 (9.17), 123.1174 (29.17), 109.1018 (48.33), 95.0863 (83.33), 93.0707 (15), 81.0707 (100), 67.0551 (100), 55.0552 (13.33) | 28.3798 | Linolenic acid | [M+H]^+^ | Fatty acid | Automatically identified | 1.00352847 | auto | part |
| 34610 | 22 | 279.2377 | 279.2382 (26.11), 277.2227 (13.89), 161.1327 (7.22), 149.0237 (55.56), 135.1173 (13.61), 123.1173 (22.5), 109.1018 (38.89), 107.0862 (20.56), 95.0863 (77.78), 93.0706 (30.56), 81.0707 (100), 79.0551 (23.61), 67.0551 (88.89), 57.0708 (18.61), 55.0552 (16.67) | 29.8449 | 9(10)-EpOME | [M-H_2_O+H]^+^ | Fatty acid | Automatically identified | 0.96166957 | auto | part |
| 37062 | 425 | 279.1762 | 149.0237 (12.14), 120.0813 (11.43), 87.1005 (6.5), 86.0972 (100), 69.0708 (12.86) | 13.0424 | Leu-Phe | [M+H]^+^ | Flavonoid | Automatically identified | 0.01294194 | auto | part |
| 194 | 22 | 277.2222 | 277.2224 (59.26), 275.1665 (11.11), 259.2108 (7.41), 201.1648 (5.93), 173.1329 (7.78), 161.1328 (10.37), 149.0236 (55.56), 135.1171 (31.11), 121.1017 (36.3), 119.086 (22.96), 109.1017 (37.04), 107.0861 (44.44), 105.0704 (27.41), 95.0862 (81.48), 93.0706 (66.67), 91.055 (22.96),81.0707 (100), 79.0551 (51.85), 67.0551 (88.89), 55.0552 (24.07) | 34.4132 | 9-Oxo-10*E*,12*Z*-octadecadienoic acid | [M-H_2_O+H]^+^ | Fatty acid | Automatically identified | 1.00059071 | auto | part |
| 23507 | 22 | 277.215 | 279.1935 (44.83), 277.178 (65.52), 149.0235 (100), 135.1169 (32.07), 121.1014 (34.48), 107.0859 (37.93), 95.086 (41.38), 93.0703 (72.41), 81.0704 (58.62), 79.0548 (48.28), 67.0549 (51.72), 57.0706 (34.48) | 29.784 | Stearidonic acid | [M+H]^+^ | Fatty acid | Automatically identified | 1.21145049 | auto | part |
| 13047 | 5 | 275.2066 | 277.1841 (100), 275.2061 (74.29), 235.1715 (28.29), 179.1062 (10.29), 147.1169 (22), 133.1013 (27.71), 119.0858 (31.43), 105.0703 (45.71), 91.0548 (28.57), 81.0705 (20.86), 69.0707 (15.14), 57.0707 (21.43) | 28.843 | 9-OxoOTrE | [M-H_2_O+H]^+^ | Fatty acid | Automatically identified | 0.50604509 | auto | part |
| 17004 | -1 | 275.2065 | 277.1835 (42.5), 275.2063 (42.5), 274.2789 (100), 256.268 (6), 159.1171 (6.5), 147.1172 (13), 145.1016 (10.5), 133.1016 (16.5), 119.086 (18.25), 105.0705 (27.5), 93.0707 (22), 81.0707 (24.25), 67.0551 (16.5), 55.0552 (13.5) | 27.7351 | 8-{(1*S*,5*R*)-4-Oxo-5-[(2*Z*)-2-penten-1-yl]-2-cyclopenten-1-yl}octanoic acid | [M-H_2_O+H]^+^ | Fatty acid | Automatically identified | 0.69633093 | auto | part |
| 17429 | 1068 | 275.0957 | 275.0961 (6.67), 191.0709 (7), 149.0601 (25.67), 145.065 (7.67), 139.0393 (100), 107.0498 (53.33) | 8.6768 | [(+)-Afzelechin](https://pubchem.ncbi.nlm.nih.gov/compound/442154) | [M+H]^+^ | Flavonoid | Automatically identified | 0.31785519 | auto | part |
| 41781 | 154 | 273.0793 | 274.0832 (15), 273.08 (76.67), 153.0183 (100), 147.0441 (45), 123.0443 (6.5), 119.0495 (18.33), 91.0549 (11.17) | 23.4952 | Naringenin | [M+H]^+^ | Flavonoid | Automatically identified | 1.18143881 | auto | part |
| 34790 | 1193 | 265.2569 | 265.2573 (88.89), 247.2453 (37.78), 223.0607 (11.33), 205.0504 (14.44), 193.0496 (28.89), 177.0549 (44.44), 135.1171 (33.33), 123.1173 (31.11), 107.0861 (51.11), 95.0861 (93.33), 93.0706 (68.89), 83.0863 (64.44), 81.0707 (91.11), 79.0549 (37.78), 69.0707 (100), 67.0551 (66.67), 57.0708 (48.89), 55.0552 (71.11) | 7.5028 | oleic acid | [M-H_2_O+H]^+^ | Fatty acid | Automatically identified | 0.85694897 | auto | part |
| 14550 | 425 | 265.1591 | 121.0846 (10), 120.0812 (100), 103.0548 (5.4), 72.0816 (17.33) | 7.5984 | Phe-Val | [M+H]^+^ | Alkaloid | Automatically identified | 0.06021382 | auto | part |
| 42133 | -1 | 263.0956 | 263.0963 (18.08), 245.0846 (7.69), 203.0703 (100), 187.0393 (11.15), 175.0392 (28.46), 145.0653 (5.38), 131.0859 (7.69), 119.0861 (8.85), 105.0703 (11.54), 93.0705 (15.77), 81.0705 (15), 67.0551 (10.38), 59.05 (13.85) | 23.9817 | trans-khellactone | [M+H]^+^ | Coumarin | Automatically identified | 0.70869667 | auto | part |
| 44250 | 1122 | 261.1638 | 261.1653 (28.33), 259.1272 (8.33), 233.1677 (9.17), 177.0552 (8.67), 121.0848 (9), 105.0705 (13.33), 91.055 (9), 86.0972 (25), 69.0707 (5.33) | 23.194 | cyclo(Phe-Leu) | [M+H]^+^ | Alkaloid | Automatically identified | 1.22587652 | auto | part |
| 10722 | 743 | 260.2016 | 260.2013 (18.08), 243.174 (7.31), 188.0706 (13.85), 170.0604 (10), 147.1129 (6.54), 132.1022 (15.77), 102.0557 (6.15), 86.0972 (30), 84.0816 (100), 56.0503 (6.92) | 4.8983 | Lys-Leu | [M+H]^+^ | Alkaloid | Automatically identified | 0.13158758 | auto | part |
| 56819 | 366 | 259.064 | 259.0647 (100), 241.0523 (6), 231.0679 (37.5), 213.0556 (30), 185.0601 (50), 163.0391 (9.5), 157.065 (7.25), 143.0608 (8), 129.0703 (27.5) | 21.5297 | 3,7,9-trihydroxy-1-methylbenzo[c]chromen-6-one | [M+H]^+^ | Flavonoid | Automatically identified | 1.23280186 | auto | part |
| 18670 | 425 | 253.1209 | 253.122 (11.84), 207.1138 (14.9), 166.0866 (57.14), 120.0813 (100), 95.0863 (5.92), 81.0706 (5.1), 69.0708 (5.1), 60.0453 (77.55) | 4.897 | Ser-Phe | [M+H]^+^ | Alkaloid | Automatically identified | 0.14893778 | auto | part |
| 37000 | 425 | 245.1891 | 87.1005 (6.86), 86.0972 (100), 69.0708 (9.14) | 10.2074 | Ile-Leu | [M+H]^+^ | Alkaloid | Automatically identified | 0.10257330 | auto | part |
| 13858 | 553 | 245.1311 | 245.1325 (60), 227.0921 (8.2), 209.0819 (19), 154.074 (28), 120.0813 (100), 115.0396 (42), 98.0608 (34), 87.0448 (12.8), 85.0656 (16), 70.066 (90),57.0709 (16.2) | 14.1747 | Phe-Pro | [M-H_2_O+H]^+^ | Alkaloid | Automatically identified | 1.12334958 | auto | part |
| 19837 | 576 | 239.2391 | 239.2396 (24.53), 209.9831 (24.53), 191.9718 (24.53), 133.0646 (22.64), 121.1013 (24.53), 109.1018 (32.08), 105.0705 (18.87), 97.102 (24.53), 95.0862 (81.13), 93.0706 (28.3), 85.102 (24.53), 83.0862 (49.06), 81.0706 (75.47), 71.0863 (45.28), 69.0708 (58.49), 67.0551 (60.38), 57.0708 (100), 55.0551 (49.06) | 7.0809 | Palmitic acid | [M+H]^+^ | Fatty acid | Automatically identified | 0.66832493 | auto | part |
| 46681 | 464 | 237.0776 | 238.082 (14), 237.0785 (100), 222.0543 (14), 207.0299 (16), 204.0428 (5.5), 193.0501 (12), 191.0342 (16), 178.0627 (5.5), 176.0474 (15), 163.0392 (5.2), 148.0523 (6.2), 147.0445 (8.5) | 17.5981 | Dimethylfraxetin | [M+H]^+^ | Coumarin | In-house library | 0.96245885 |  |  |
| 3650 | 464 | 237.0777 | 237.0757 (100), 222.0521 (12.5), 207.0287 (18.33), 193.0497 (13.33), 191.0336 (14.17), 178.0628 (6.25), 147.044 (6.92) | 17.8074 | 5,6,7-Trimethoxy- coumarin | [M+H]^+^ | Coumarin | In-house library | 0.68392314 |  |  |
| 21466 | 425 | 231.1733 | 86.0972 (5.79), 72.0816 (100), 55.0552 (11.05) | 6.5858 | Val-Leu | [M+H]^+^ | Alkaloid | Automatically identified | 0.04786878 | auto | part |
| 56918 | -1 | 233.1529 | 233.1073 (83.33), 232.0992 (55), 231.0919 (46.11), 219.0918 (12.22), 187.1441 (8.33), 132.1022 (16.67), 118.0655 (23.33), 86.097 (23.89), 74.0607 (100), 56.0503 (15) | 4.4597 | Thr-Leu | [M+H]^+^ | Alkaloid | Automatically identified | 1.21387125 | auto | part |
| 37044 | 425 | 231.173 | 116.0711 (17.14), 86.0972 (55), 72.0816 (100), 70.066 (6.79), 55.0552 (12.14) | 5.5273 | Val-Ile | [M+H]^+^ | Alkaloid | Automatically identified | 0.08392540 | auto | part |
| 37043 | 425 | 231.1724 | 116.0711 (10), 87.1005 (6.88), 86.0972 (100), 72.0816 (30), 70.066 (10), 69.0708 (10.62) | 4.7306 | Leu-Val | [M+H]^+^ | Alkaloid | Automatically identified | 0.07459561 | auto | part |
| 356 | -1 | 231.1611 | 233.1554 (48.78), 217.1449 (85.37), 187.1117 (10.73), 171.1385 (11.22), 157.101 (11.22), 145.1013 (22.44), 139.1119 (97.56), 131.0857 (36.59), 121.1015 (92.68), 107.0861 (34.15), 105.0705 (34.15), 95.086 (51.22), 81.0706 (68.29), 69.0707 (100), 55.0551 (51.22) | 24.9834 | Dimethyl sebacate | [M+H]^+^ | Fatty acid | Automatically identified | 0.99114826 | auto | part |
| 37142 | 425 | 229.1568 | 229.1577 (14.71), 227.1775 (16.47), 205.9888 (5.1), 156.0815 (6.08), 116.0712 (54.9), 114.0918 (7.84), 96.0815 (9.8), 86.0972 (100), 72.0817 (27.45), 70.066 (70.59), 69.0708 (17.65) | 4.2492 | Leu-Pro | [M+H]^+^ | Alkaloid | Automatically identified | 1.21803240 | auto | part |
| 13942 | 140 | 229.1457 | 229.1455 (95), 193.1221 (10), 175.112 (28), 157.1016 (34.5), 147.1171 (100), 133.1015 (18.5), 119.0861 (39.5), 107.0862 (20), 95.0862 (17.5), 91.055 (48.5), 81.0707 (75), 79.0551 (28), 69.0708 (24), 67.0551 (43), 55.0551 (21) | 24.1147 | Traumatic Acid | [M+H]^+^ | Fatty acid | Automatically identified | 1.34918887 | auto | part |
| 34567 | 283 | 223.0615 | 223.062 (100), 207.03 (10), 190.0264 (24.74), 179.034 (7.37), 163.0392 (12.11), 162.0314 (25.26), 135.0449 (6.32), 107.0498 (20.53) | 12.2826 | Isofraxidin | [M+H]^+^ | Coumarin | Automatically identified | 0.37031879 | auto | part |
| 41644 | 993 | 221.0458 | 221.0463 (100), 219.1037 (6.38), 206.0222 (5.51), 189.091 (5.36), 174.0676 (5.22), 163.0392 (23.19), 135.0444 (28.99), 107.0498 (11.88) | 16.8064 | Gallic Acid Ethyl Ester | [M+Na]^+^ | Benzene derivative | Automatically identified | 0.49689830 | auto | part |
| 23956 | 179 | 220.1193 | 220.1193 (33.33), 202.1073 (25.83), 184.0966 (17.5), 124.0759 (16.67), 98.0242 (15), 90.0555 (100), 72.0451 (20), 57.0708 (10) | 3.646 | Pantothenic acid | [M+H]^+^ | Alkaloid | Automatically identified | 0.88046929 | auto | part |
| 415 | -1 | 217.1451 | 219.1759 (100), 217.1616 (39.36), 201.1643 (21.28), 161.0961 (25.53), 159.1169 (36.17), 145.1011 (25.53), 139.1117 (43.62), 133.1015 (26.6), 121.1015 (68.09), 107.086 (29.79), 105.0703 (42.55), 95.0861 (42.55), 93.0705 (46.81), 81.0706 (58.51), 69.0707 (53.19), 67.0551 (34.04), 55.0551 (30.85) | 24.9712 | Sebacic acid | [M+H]^+^ | Fatty acid | Automatically identified | 0.99419933 | auto | part |
| 12670 | 478 | 209.0817 | 209.0821 (50), 207.0663 (61.25), 191.0706 (27.5), 177.055 (66.25), 151.0757 (23.75), 149.0601 (46.25), 131.0495 (16.25), 121.0653 (38.75), 107.0499 (17.5), 103.0549 (33.75), 91.0549 (37.5), 55.0188 (100) | 13.574 | Sinapoyl aldehyde | [M+H]^+^ | Benzene derivative | Automatically identified | 0.58975991 | auto | part |
| 30768 | 952 | 209.0817 | (43.66), 207.0663 (67.61), 191.1434 (29.58), 177.0551 (57.75), 153.0912 (21.13), 151.0757 (30.99), 149.0601 (100), 137.0602 (16.9), 121.0653 (88.73), 109.0654 (42.25), 107.0861 (30.99), 106.042 (70.42), 95.0499 (32.39), 93.0706 (32.39), 91.055 (78.87), 81.0707 (22.54), 67.0551 (15.49), 55.0188 (28.17) | 22.0128 | Genipin | [M-H_2_O+H]^+^ | Terpenoid | Automatically identified | 0.92684701 | auto | part |
| 23293 | 689 | 209.0453 | 209.0455 (100), 194.0213 (21.05), 181.0498 (5.79), 163.0393 (11.05), 149.0237 (25.26), 135.0444 (8.42), 107.0498 (10) | 8.6281 | Fraxetin | [M+H]^+^ | Coumarin | Automatically identified | 0.41789584 | auto | part |
| 12669 | 1150 | 207.0661 | 207.0659 (100), 191.0341 (12.14), 179.0703 (9.29), 163.0391 (8.57), 151.0755 (28.57) | 12.9726 | [Jasminoside](https://pubchem.ncbi.nlm.nih.gov/compound/23786444) | [M+H]^+^ | Benzene derivative | Automatically identified | 1.02540174 | auto | part |
| 62459 | 539 | 207.0661 | 207.0659 (100), 191.0338 (8.95), 175.039 (63.16), 163.039 (6.84), 147.0441 (51.58), 119.0495 (63.16), 91.0548 (68.42) | 29.659 | [Sinapinic acid](https://pubchem.ncbi.nlm.nih.gov/compound/637775) | [M-H_2_O+H]^+^ | Cinnamic acid | Automatically identified | 0.49152599 | auto | part |
| 36965 | 141 | 205.098 | 207.0661 (8.03), 189.0745 (14.75), 188.071 (91.8), 170.0603 (7.87), 159.0921 (10.82), 147.0639 (11.15), 146.0604 (100), 144.0812 (21.31), 132.0813 (11.15), 118.0657 (45.9) | 4.9553 | [D-Tryptophan](https://pubchem.ncbi.nlm.nih.gov/compound/9060) | [M+H]^+^ | Alkaloid | Automatically identified | 0.10469815 | auto | part |
| 37544 | -1 | 197.1291 | 197.1294 (100), 181.0499 (27.78), 169.1339 (27.78), 151.0395 (62.96), 140.0471 (20.37), 125.0602 (24.07), 109.0655 (12.78), 95.0498 (18.52), 81.0707 (12.04), 79.055 (13.52), 70.066 (75.93), 67.0551 (12.22), 55.0552 (11.67) | 6.9713 | Cyclo(*L*-Val-L-Pro) | [M+H]^+^ | Alkaloid | Automatically identified | 1.18544331 | auto | part |
| 66211 | 835 | 197.1176 | 197.1173 (74), 179.1067 (100), 161.0962 (32), 151.112 (6.2), 136.12 (6.6), 135.117 (52), 133.1014 (68), 119.0858 (9.2), 107.0859 (66), 93.0704 (24), 81.0703 (5.6), 67.0548 (5.8) | 13.0527 | Loliolide | [M+H]^+^ | Terpenoid | Automatically identified | 1.13515306 | auto | part |
| 19207 | 894 | 195.0656 | 195.0877 (41.54), 177.0547 (100), 163.0391 (7.69), 149.0598 (21.54), 145.0285 (71.54), 117.0339 (43.85), 89.0392 (23.08) | 18.0993 | [Ferulic acid](https://pubchem.ncbi.nlm.nih.gov/compound/445858) | [M+H]^+^ | Cinnamic acid | Automatically identified | 1.68952098 | auto | part |
| 66236 | 894 | 195.0655 | 195.0877 (68.24), 177.0547 (100), 163.0388 (8.82), 149.0597 (21.18), 145.0285 (77.65), 135.0444 (7.06), 117.0338 (43.53), 91.0547 (5.18), 89.0392 (21.18) | 18.2643 | 3-Hydroxy-4-methoxycinnamic acid | [M+H]^+^ | Cinnamic acid | Automatically identified | 1.575790746 | auto | part |
| 41633 | 396 | 188.0709 | 188.0708 (23.5), 170.0605 (9), 147.0639 (10.5), 146.0603 (100), 144.0812 (23), 143.0733 (11.5), 142.0657 (5.5), 119.0691 (5.5), 118.0657 (70), 115.0548 (6) | 4.2606 | Abrine | [M-CH3NH_2_+H]^+^ | Alkaloid | Automatically identified | 0.29217410 | auto | part |
| 30622 | 35 | 185.0813 | 185.0811 (32.84), 169.0499 (17.91), 154.0628 (37.31), 153.0549 (100), 141.0547 (9.4), 139.0393 (17.91), 127.0395 (6.87), 125.0601 (73.13), 111.045 (8.81), 95.0134 (6.87) | 7.5354 | 3,4,5-Trimethoxyphenol | [M+H]^+^ | Benzene derivative | Automatically identified | 0.36138297 | auto | part |
| 8188 | 501 | 183.0656 | 183.0658 (8.19), 155.0706 (22.34), 153.0548 (10.64), 123.0445 (84.04), 95.0498 (100), 71.05 (9.89), 67.0551 (10.64), 53.0395 (15.96) | 10.2905 | Syringaldehyde | [M+H]^+^ | Benzene derivative | Automatically identified | 0.72676631 | auto | part |
| 50757 | -1 | 181.1226 | 181.0499 (53.62), 163.0393 (24.64), 147.0443 (100), 135.0443 (20.29), 121.0653 (18.84), 119.0496 (50.72), 107.0498 (10.58), 91.0549 (43.48), 79.055 (21.74), 55.0187 (9.42) | 24.4028 | Dihydroactinidiolide | [M+H]^+^ | Terpenoid | Automatically identified | 1.06806836 | auto | part |
| 56804 | 522 | 179.0706 | 179.0706 (100), 161.0601 (13.33), 133.0652 (11.33), 119.0496 (5.07), 105.0706 (12) | 13.3748 | Coniferaldehyde | [M+H]^+^ | Benzene derivative | Automatically identified | 1.21197904 | auto | part |
| 8260 | 117 | 179.0706 | 148.0475 (11.46), 147.0441 (100), 119.0494 (37.5), 91.0547 (18.12) | 6.6401 | Methoxycinnamic acid | [M+H]^+^ | Cinnamic acid | Automatically identified | 0.22204641 | auto | part |
| 36958 | 193 | 179.0342 | 180.0375 (10.2), 179.0342 (100), 151.0392 (7.55), 133.0288 (26.53), 123.0445 (38.78) | 6.8036 | Esculetin | [M+H]^+^ | Coumarin | Automatically identified | 0.31102803 | auto | part |
| 57083 | 149 | 177.1641 | 177.1638 (82), 145.0285 (34), 135.1169 (26), 121.1015 (100), 119.0858 (30), 107.086 (76), 105.0703 (36), 95.0861 (76), 93.0704 (60), 91.0548 (40), 81.0705 (50), 79.0549 (30), 69.0707 (30), 55.0551 (22) | 27.4365 | [Dihydro-alpha-ionone](https://pubchem.ncbi.nlm.nih.gov/compound/35821) | [M+H]^+^ | Terpenoid | Automatically identified | 0.92914370 | auto | part |
| 62452 | 165 | 175.1231 | 145.0843 (12.17), 144.0808 (100), 132.0809 (21.52) | 5.2312 | *[N](https://pubchem.ncbi.nlm.nih.gov/compound/6088)*[-Methyltryptamine](https://pubchem.ncbi.nlm.nih.gov/compound/6088) | [M+H]^+^ | Alkaloid | Automatically identified | 0.85634788 | auto | part |
| 28677 | 1331 | 173.0966 | 173.0959 (100), 158.0727 (49.38), 155.0856 (24.38), 145.1012 (47.5), 131.0859 (9.38), 117.0702 (7.5), 105.0702 (11.25), 72.0451 (7.5) | 20.324 | [gamma-Tocotrienol](https://pubchem.ncbi.nlm.nih.gov/compound/5282349) | [M+H]^+^ | Benzene derivative | Automatically identified | 1.01488386 | auto | part |
| 17134 | 183 | 171.1383 | 173.1326 (100), 171.117 (16.95), 153.1277 (47.46), 135.1172 (61.02), 131.0858 (52.54), 127.0395 (22.03), 109.1018 (37.29), 107.0861 (88.14), 97.0655 (72.88), 95.0862 (37.29), 93.0706 (57.63), 81.0707 (20.34), 69.0707 (54.24), 67.0551 (20.34), 55.0552 (18.64) | 15.6524 | 10-Hydroxydecanoic acid | [M+H]^+^ | Fatty acid | Automatically identified | 1.28329637 | auto | part |
| 23516 | 425 | 166.0866 | 121.0844 (10), 120.0811 (100), 103.0547 (8.46) | 3.0081 | Phenylalanine | [M+H]^+^ | Alkaloid | Automatically identified | 1.33955888 | auto | part |
| 8546 | 149 | 165.055 | 165.0551 (40.91), 147.0444 (100), 135.0444 (18.18), 123.0446 (20), 119.0497 (54.55), 107.0861 (21.82), 91.055 (44.55), 79.055 (16.36), 67.0551 (10.91) | 7.2019 | *L*-Tyrosine | [M-NH_3_+H]^+^ | Alkaloid | Automatically identified | 1.25680784 | auto | part |
| 12800 | 176 | 163.0393 | 163.0392 (86.9), 145.0287 (44.05), 135.0444 (100), 117.034 (59.52), 107.0497 (14.29), 89.0393 (69.05), 79.055 (10.95) | 7.0962 | Caffeic acid | [M-H_2_O+H]^+^ | Cinnamic acid | Automatically identified | 1.00310462 | auto | part |
| 23759 | -1 | 161.0964 | 161.096 (25.97), 159.1575 (100), 133.1012 (16.88), 119.0858 (32.47),105.0702 (48.05), 103.095 (32.47), 102.0917 (27.27), 91.0547 (20.78), 57.0706 (18.18) | 28.0399 | Pinolenic acid | [M+H]^+^ | Fatty acid | Automatically identified | 0.80491868 | auto | part |
| 3816 | 259 | 161.0964 | 161.0961 (100), 133.1012 (37.04), 131.0858 (27.78), 119.0858 (37.04), 105.0702 (70.37), 91.0547 (27.78), 79.0548 (7.59) | 25.2986 | 9,12-Octadecadiynoic Acid | [M+2Na]^+^ | Fatty acid | Automatically identified | 0.98675248 | auto | part |
| 66103 | 34 | 385.1133 | 223.0602 (56.14), 205.0497 (100), 121.065 (6.49), 121.0287 (7.37), 85.029 (6.14) | 11.0071 | Calycanthoside | [M+H]^+^ | Coumarin | In-house library | 0.97128585 |  |  |
| 25332 | 50 | 487.144 | 325.0918 (8.06), 307.0831 (6.13), 211.0601 (100), 193.0496 (67.74), 185.0447 (22.26), 169.0496 (35.48), 165.0547 (16.77), 147.0441 (15.48), 133.0285 (41.94), 115.0393 (18.71), 109.0287 (10), 97.0289 (6.45), 85.0289 (12.9), 81.034 (6.77), 69.0341 (10.32) | 6.1939 | Xeroboside | [M+H]^+^ | Coumarin | In-house library | 1.44074291 |  |  |
| 30838 | 50 | 503.1394 | 501.1612 (11), 227.0551 (100), 209.0446 (75), 185.0446 (60), 181.0495 (18.5), 163.0391 (13.5), 149.0236 (22), 139.0391 (11), 135.0441 (8), 125.0234 (14.5), 97.0289 (5.5), 85.0289 (7) | 7.1478 | Nitensoside A | [M+H]^+^ | Coumarin | In-house library | 0.50347608 |  |  |
| 13190 | 31 | 547.1674 | 194.0534 (11.89), 193.05 (100), 178.0266 (6.79), 133.0287 (13.4) | 7.1406 | Nitensoside B | [M+H]^+^ | Coumarin | In-house library | 0.53141818 |  |  |
| 3694 | 107 | 383.0761 | 383.0759 (100), 327.0863 (33.33), 299.0912 (9.17), 267.0646 (8.33) | 24.5984 | Hymenain | [M+H]^+^ | Coumarin | In-house library | 0.44008918 |  |  |
| 4248 | 107 | 413.0867 | 413.0866 (100), 353.0652 (12.07), 325.0712 (7.59), 297.0756 (17.24), 281.0441 (6.55), 265.0499 (6.21) | 19.8449 | Chimsalicifoliusin A | [M+H]^+^ | Coumarin | In-house library | 0.26327902 |  |  |
| 3946 | 107 | 443.0975 | 443.0973 (100), 387.108 (15.49), 355.0808 (5.92), 327.0864 (10.7), 299.0899 (5.35), 218.0572 (5.21) | 25.0745 | Arteminorin A | [M+H]^+^ | Coumarin | In-house library | 0.44228214 |  |  |
| 35365 | 107 | 443.1141 | 221.0463 (12.92), 209.0455 (100), 208.0698 (8.33), 207.0663 (69.44), 179.0707 (5.83), 163.0393 (9.03), 153.055 (16.67), 151.0755 (6.81), 135.0442 (5.42), 125.0602 (7.64) | 22.5012 | 3,3'-Biisofraxidin | [M+H]^+^ | Coumarin | In-house library | 0.55477954 |  |  |
| 4036 | 107 | 383.0758 | 383.0759 (100), 207.0291 (11.82), 191.0336 (30.91), 177.0547 (86.36), 163.0392 (29.09), 149.0232 (29.09), 133.0285 (24.55), 123.0442 (22.73), 121.0288 (13.64), 107.0495 (23.64), 95.0496 (33.64), 79.0548 (16.36) | 18.4481 | Chimsalicifoliusin B | [M+H]^+^ | Coumarin | In-house library | 0.26051544 |  |  |
| 870 | 107 | 397.1061 | 397.1054 (100), 205.0506 (11.67), 193.05 (77.78), 177.055 (20.56), 165.0549 (31.67), 149.0607 (8.33), 135.0441 (8.89), 121.0651 (5.56), 107.0496 (8.33), 79.0549 (8.33) | 29.5478 | [2-[(*E*)-3-Oxo-3-phenylprop-1-enyl]phenyl] 2-oxo chromene-3-carboxylate | [M+H]^+^ | Coumarin | Pubchem | 1.06036442 |  |  |
| 23618 | 107 | 429.0815 | 429.0815 (100), 401.0847 (8.89), 383.0752 (14.44), 373.0906 (17.78), 341.0654 (6.67), 313.0704 (10), 285.0751 (6.67) | 22.741 | [Genistein 7-](https://pubchem.ncbi.nlm.nih.gov/compound/139584229)*[O](https://pubchem.ncbi.nlm.nih.gov/compound/139584229)*[-alpha-4'-anhydro-4',5'-didehydroglucuronide](https://pubchem.ncbi.nlm.nih.gov/compound/139584229) | [M+H]^+^ | Flavonoid | Pubchem | 0.69638215 |  |  |
| 217 | 107 | 603.1366 | 397.1063 (100), 219.067 (8.45), 205.0503 (8.73), 193.0501 (39.09), 179.0343 (100), 177.0548 (12.73), 165.0551 (12.73), 151.0395 (13.64), 135.0446 (8.55) | 29.5416 | [Eriodictyol 7-(6-galloylglucoside)](https://pubchem.ncbi.nlm.nih.gov/compound/74819398) | [M+H]^+^ | Flavonoid | Pubchem | 1.08464248 |  |  |
| 3958 | 45 | 387.1073 | 387.107 (100), 337.0706 (8.65), 263.0548 (18.46), 233.0449 (8.46), 207.0289 (38.46), 179.0339 (23.08), 163.0392 (9.23), 147.0441 (15.38), 137.0598 (25), 135.0441 (13.46), 131.0493 (30.77), 123.0442 (13.27), 107.0492 (5.38), 91.0546 (9.42), 79.0546 (5.38) | 21.1781 | Cleomiscosin B | [M+H]^+^ | Coumarin | In-house library | 0.20976537 |  |  |
| 23437 | 45 | 387.1071 | 387.1068 (35.48), 369.0957 (12.9), 337.0706 (70.97), 207.0289 (21.61), 179.034 (15.48), 162.0676 (100), 161.0599 (23.87), 147.0441 (45.16), 131.0494 (61.29), 103.0548 (10.32), 95.0496 (10.32), 91.0546 (8.71) | 17.4694 | Cleomiscosin A | [M+H]^+^ | Coumarin | In-house library | 0.78295968 |  |  |
| 3970 | 45 | 417.1179 | 417.1152 (16.59), 367.0812 (100), 207.0288 (16.82), 181.0497 (15.45), 179.0341 (11.82), 167.0704 (27.27), 161.0597 (63.64), 132.0572 (90.91), 131.0493 (81.82), 105.0703 (16.82), 95.0496 (14.09), 91.0547 (7.27) | 21.7519 | Cleomiscosin C | [M+H]^+^ | Coumarin | In-house library | 0.24019743 |  |  |
| 9357 | 45 | 429.1345 | 369.1096 (23.75), 337.0797 (70.83), 245.0481 (29.17), 205.0507 (41.67), 179.0339 (12.5), 163.0394 (13.75), 162.0679 (100), 161.06 (37.92), 147.0443 (45.83), 137.06 (20.42), 131.0496 (91.67), 119.0497 (24.17), 103.055 (25), 95.05 (9.17), 91.0549 (20.42) | 25.4424 | (*E*)-3-[4-[[7-Hydroxy-3-(4-methoxyphenyl)-2-oxochromen-4-yl]methyl]phenyl]prop-2-enoic acid | [M+H]^+^ | Coumarin | Pubchem | 0.47352911 |  |  |
| 15141 | 355 | 603.1003 | 603.0989 (16.67), 585.0847 (5.19), 313.0414 (44.44), 303.0566 (85.19), 301.0408 (28.52), 287.0607 (11.85), 273.0437 (100), 257.0476 (6.3), 245.0478 (18.89), 217.0508 (24.44), 189.0552 (5.56), 179.0347 (6.3), 153.0183 (24.81), 137.0233 (12.59), 121.0289 (5.93) | 25.7491 | Chimsalicifoliusin C | [M+H]^+^ | Coumarin | In-house library | 0.54283343 |  |  |
| 42162 | 355 | 587.1046 | 587.1072 (16.67), 313.0414 (47.62), 303.056 (11.9), 301.0415 (7.62), 287.0604 (66.67), 273.0437 (14.76), 257.0477 (100), 245.048 (13.33), 229.0517 (11.9), 217.0509 (12.86), 179.0343 (5.71), 153.018 (16.19), 147.0443 (7.14), 121.0289 (11.43), 93.0704 (5.71) | 27.7164 | [(2*S*,3*R*,4*S*,5*S*)-2-[2-(3,4-Dihydroxyphenyl)-5,7-dihydroxy-4-oxochromen-3-yl]oxy-4,5-dihydroxyoxan-3-yl] 3,4,5-trihydroxybenzoate | [M+H]^+^ | Flavonoid | Pubchem | 0.56037169 |  |  |
| 23312 | 31 | 355.1022 | 194.0531 (12), 193.0497 (100), 178.0262 (11), 133.0285 (18) | 7.7963 | Chlorogenic acid | [M+H]^+^ | Cinnamic acid | In-house library | 1.14197465 |  |  |
| 50734 | 12 | 339.1062 | 339.1053 (100), 227.1753 (15.8), 209.165 (36), 177.0526 (15.4), 147.0445 (6.2), 113.1076 (22), 79.0549 (7.4), 69.0706 (19.4) | 13.4604 | *p*-Coumaroylquinic acid | [M+H]^+^ | Cinnamic acid | In-house library | 0.69914770 |  |  |
| 933 | 12 | 341.1138 | 341.2614 (7), 339.1053 (100), 322.2491 (54), 227.1753 (15.8), 209.165 (36), 177.0526 (15.4), 147.0445 (6.2), 114.0917 (56), 113.1076 (22), 96.0813 (48), 79.0549 (7.4), 69.0706 (19.4) | 10.3713 | Linocinnamarin | [M+H]^+^ | Cinnamic acid | In-house library | 1.22537138 |  |  |
| 66785 | 12 | 293.1005 | 293.1024 (10.83), 233.0813 (7.25), 219.0654 (10.83), 205.0866 (6.25), 179.0704 (82.5), 163.0753 (48.33), 151.0391 (75.83), 133.0649 (37.5), 127.0392 (100), 103.0546 (19.17), 97.0288 (16.67), 69.0341 (35), 55.0186 (15.83) | 14.8226 | [Altenuene](https://pubchem.ncbi.nlm.nih.gov/compound/34687) | [M+H]^+^ | Coumarin | Pubchem | 0.20541267 |  |  |
| 31430 | 12 | 309.0727 | 309.1665 (5.38), 247.0598 (7.54), 193.0496 (73.85), 167.034 (100), 147.0441 (27.69), 111.0444 (10.77), 85.0289 (7.23) | 16.8825 | 4-Hydroxy-3-[(*E*)-3-(4-hydroxyphenyl)prop-2-enoyl]chromen-2-one | [M+H]^+^ | Coumarin | Pubchem | 0.62964197 |  |  |
| 50963 | 12 | 325.0916 | 165.0548 (6.73), 163.039 (60), 147.0441 (100), 135.0441 (10.91), 119.0494 (21.82), 97.0288 (5.45), 91.0547 (11.45), 69.0341 (9.45) | 9.2647 | [7-Hydroxycoumarin glucoside](https://pubchem.ncbi.nlm.nih.gov/compound/99693) | [M+H]^+^ | Coumarin | Pubchem | 0.45848219 |  |  |
| 66120 | 50 | 371.1322 | 373.182 (6.5), 371.1697 (41), 371.1313 (100), 209.0785 (26), 185.042 (11), 114.0916 (5.5) | 13.9069 | Methyl 4-*β*-*D*- glucopyranosyl -ferulate | [M+H]^+^ | Cinnamic acid | In-house library | 1.14163004 |  |  |
| 57007 | 609 | 367.1516 | 206.0905 (13.85), 205.0871 (100), 85.0292 (5.38) | 23.7542 | Cassia *cis-trans* diphenylpropanoid | [M+H]^+^ | Cinnamic acid | In-house library | 1.23248332 |  |  |
| 57123 | 609 | 379.151 | 218.0911 (14.62), 217.0878 (100), 114.0919 (6.92), 85.0292 (9.23) | 27.1594 | 4-[4-(4-Ethylphenoxy)butoxy]furo[3,2-g]chromen-7-one | [M+H]^+^ | Coumarin | Pubchem | 1.24792886 |  |  |
| 4743 | -1 | 393.2074 | 394.2097 (30.91), 393.249 (6.64), 393.2084 (100) | 11.4578 | Grammatin A | [M+H]^+^ | Flavonoid | In-house library | 0.45369606 |  |  |
| 31615 | -1 | 301.1044 | 303.0502 (100), 301.1404 (22.35), 301.0343 (7.88), 169.0133 (10.71), 121.0287 (9.53) | 13.4621 | 5-Hydroxy-7,4’-dimethoxyflavone | [M+H]^+^ | Flavonoid | In-house library | 1.25001948 |  |  |
| 42296 | 790 | 271.1001 | 273.1435 (100), 271.1017 (64.52), 257.1106 (19.35), 241.089 (6.45), 211.0765 (31.94), 197.0602 (8.39), 183.0809 (31.61), 165.0703 (58.06), 153.0702 (29.35), 119.0497 (14.19), 91.055 (27.42), 67.0551 (13.55) | 10.4232 | (-)-Pinostrobin | [M+H]^+^ | Flavonoid | In-house library | 1.10521201 |  |  |
| 22911 | 1210 | 345.1075 | 196.0325 (10.54), 195.0291 (100), 85.0291 (24.59), 57.0344 (9.19) | 18.9484 | 7,3’,4’-Trimethylquercetin | [M+H]^+^ | Flavonoid | In-house library | 0.32951752 |  |  |
| 13069 | 31 | 457.113 | 209.0453 (100), 181.0503 (6.55), 163.0395 (6.9), 149.0236 (8.28), 85.029 (5.17), 70.066 (5.17) | 10.2493 | [Epicatechin 3-](https://pubchem.ncbi.nlm.nih.gov/compound/467296)*[O](https://pubchem.ncbi.nlm.nih.gov/compound/467296)*[-(3-](https://pubchem.ncbi.nlm.nih.gov/compound/467296)*[O](https://pubchem.ncbi.nlm.nih.gov/compound/467296)*[-methylgallate)](https://pubchem.ncbi.nlm.nih.gov/compound/467296) | [M+H]^+^ | Flavonoid | Pubchem | 0.45533121 |  |  |

Appendix I

Source codes in R language for screening and identification of compounds in *C. salicifolius.*

# Set Working Directory and import data

rm(list = ls())

setwd("C:/Users/Administrator/Desktop/mass/data3/")

data_all<-read.csv("XLM_dif_time_neg_mgf_converted.csv",header = TRUE,sep = ",") # the mass data converted by MZmine soft and MsBackendMgf package

DPI_all<-read.csv("DPI.csv",header = TRUE,sep = ",") # Characteristic ions of the compounds in this work

HNL<-read.csv("HNL.csv",header = TRUE,sep = ",") # Neutral loss ions of the compounds in this work

# import R package

library(dplyr)

##########################################################

#########################################################

########################################################

# (1) Automatically screen and identify flavonoids, cinnamic acids, benzoic acids and quinic acids in Calycanthus chinensis

compound_identified<-function(data,DPI,HNL){

cp<-data.frame()

name<-unique(DPI$Name)

for (i in (1:length(name))){

df<-DPI[which(DPI$Name==name[i]),]

data$label<-0

for (j in (1:nrow(df))){

data$label[which(abs(data$MS2-df$DPI_MW[j])<0.005)]<-df$DPI_No[j]

}

tf<-data[which(data$label!=0),]

frequency <- tf %>% count(Num)

frequency$score<-round(frequency$n/nrow(df),2)

frequency$Name<-df$Name[1]

cp<-rbind(cp,frequency)

}

cp<-cp[which(cp$score>=0.6),]

MS1 <- DPI[grepl("\\_1", DPI$DPI_No), ]

cp <- inner_join(cp, MS1[,c("Name","Chemical_class","DPI_MW")], by = "Name")

cp <- inner_join(cp, unique(data[,c("Num","MS1")]), by = "Num")

cp$HNL<-0

cp$HNL<-cp$MS1-cp$DPI_MW

cp$HNL_iden<-0

for (i in (1:nrow(HNL))){

cp$HNL_iden[which(abs(cp$HNL-HNL$NL_MW[i])<0.005)]<-HNL$Name[i]

}

cp$identified<-0

sp<-cp[which(abs(cp$HNL)<0.005),]

sp$identified<-sp$Name

tp<-cp[which(cp$HNL_iden!=0),]

tp$identified<-paste(tp$Name,tp$HNL_iden,sep="+")

cp<-cp[-which(abs(cp$HNL)<0.005 | cp$HNL_iden!=0),]

cp<-rbind(sp,tp,cp)

return(cp)

}

data<-data_all

HNL<-HNL

DPI<-DPI_all[which(DPI_all$Chemical_class!=c("Coumarins")),]

result<-compound_identified(data,DPI,HNL)

write.csv (result,"XLM_neg_results_1.csv", row.names=F)

##########################################################

#########################################################

########################################################

# (2) Automatically screen and identify coumarins

compound_identified_2<-function(data,DPI,HNL){

## (2.1) coumarins screened by DPI

data$DPI_No<-0

for (i in (1:nrow(DPI))){

data$DPI_No[which(abs(data$MS1-DPI$DPI_MW[i])<0.005)]<-DPI$DPI_No[i]

data$DPI_No[which(abs(data$MS2-DPI$DPI_MW[i])<0.005)]<-DPI$DPI_No[i]

}

node_DPI<-unique(data$Num[which(data$DPI_No!=0)]) #

cp<-unique(data[which(data$DPI_No!=0),c("Num","MS1","DPI_No")])

## (2.2) Calculate neutral loss ions of CO and CH3 for further screening of coumarins

## The calculation ideas of neutral loss ions refer to the following literature

###############################################################################################

# Shipei Xing, Yan Hu, Zixuan Yin, Min Liu, Xiaoyu Tang, Mingliang Fang, and Tao Huan. #

# Retrieving and Utilizing Hypothetical Neutral Losses from Tandem Mass Spectra for Spectral #

# Similarity Analysis and Unknown Metabolite Annotation.Analytical Chemistry 2020 92 (21), #

# 14476-14483. DOI: 10.1021/acs.analchem.0c02521 #

###############################################################################################

data<-data[which(data$Num %in% node_DPI),]

data$dif<-abs(as.numeric(data$MS1)-as.numeric(data$MS2))*1000000/as.numeric(data$MS1)

test<-data[which(data$dif<20),c(1:6)]

df<-data[which(data$Num %in% unique(test$Num)==FALSE),]

df<-unique(df[,c("Num","RT","MS1")])

df$MS2<-df$MS1

df$MS2Int<-0

df$R_abudance<-0

test<-rbind(test,df)

data<-data[,c(1:6)]

test<-rbind(test,data)

test<-unique(test)

result <- test %>% count(Num)

Node<-result$Num[which(result$n>1)]

test<-test[which(test$Num %in% Node),]

ID<-unique(test$Num)

HNL_function<-function(y){

vec <-y[which(test$Num==ID[1]),c("MS2","R_abudance","Num")]

vec <- arrange(vec, desc(MS2))

result1 <- combn(vec$MS2, 2, FUN = function(x) x[1] - x[2])

result2 <- combn(vec$R_abudance, 2, FUN = function(x) (x[1] + x[2])/2)

result_df1 <- data.frame(

MS2_1 = vec$MS2[combn(seq_along(vec$MS2), 2, FUN = function(x) x[1])],

MS2_2 = vec$MS2[combn(seq_along(vec$MS2), 2, FUN = function(x) x[2])],

HNL = result1,

R_abudance = result2

)

result_df1$Num<-ID[1]

HNL<-result_df1

for (i in 2:length(ID)){

vec <-y[which(test$Num==ID[i]),c("MS2","R_abudance","Num")]

vec <- arrange(vec, desc(MS2))

result1 <- combn(vec$MS2, 2, FUN = function(x) x[1] - x[2])

result2 <- combn(vec$R_abudance, 2, FUN = function(x) (x[1] + x[2])/2)

result_df2 <- data.frame(

MS2_1 = vec$MS2[combn(seq_along(vec$MS2), 2, FUN = function(x) x[1])],

MS2_2 = vec$MS2[combn(seq_along(vec$MS2), 2, FUN = function(x) x[2])],

HNL = result1,

R_abudance = result2

)

result_df2$Num<-ID[i]

HNL<-rbind(HNL,result_df2)

}

return(HNL)

}

HNL_list<-HNL_function(test)

CO<-unique(HNL_list$Num[which(abs(HNL_list$HNL-27.9949)<0.005)])

CH3<-unique(HNL_list$Num[which(abs(HNL_list$HNL-15.0235)<0.005)])

node_HNL<-CO[which(CO %in% CH3)]

# (2.3) coumarins screened and identified by DPI and HNL

cp<-cp[which(cp$Num %in% node_HNL),]

cp <- inner_join(cp, DPI[,c("DPI_No","DPI_MW")], by = "DPI_No")

cp$HNL<-cp$MS1-cp$DPI_MW

cp$HNL_iden<-0

for (i in (1:nrow(HNL))){

cp$HNL_iden[which(abs(cp$HNL-HNL$NL_MW[i])<0.005)]<-HNL$Name[i]

}

cp$identified<-0

sp<-cp[which(abs(cp$HNL)<0.005),]

sp$identified<-sp$DPI_No

tp<-cp[which(cp$HNL_iden!=0),]

tp$identified<-paste(tp$DPI_No,tp$HNL_iden,sep="+")

cp<-cp[-which(abs(cp$HNL)<0.005 | cp$HNL_iden!=0),]

cp<-rbind(sp,tp,cp)

return(cp)

}

DPI<-DPI_all[which(DPI_all$Chemical_class==c("Coumarins")),]

data<-data_all

HNL<-HNL

result2<-compound_identified_2(data,DPI,HNL)

write.csv (result2,"XLM_neg_results_2.csv", row.names=F)
